# Characterization of SARS-CoV-2 proteins reveals Orf6 pathogenicity, subcellular localization, host interactions and attenuation by Selinexor

**DOI:** 10.1186/s13578-021-00568-7

**Published:** 2021-03-25

**Authors:** Jin-Gu Lee, Weiliang Huang, Hangnoh Lee, Joyce van de Leemput, Maureen A. Kane, Zhe Han

**Affiliations:** 1 Center for Precision Disease Modeling, Department of Medicine, University of Maryland School of Medicine, Baltimore, MD USA; 2 Division of Endocrinology, Diabetes and Nutrition, Department of Medicine, University of Maryland School of Medicine, Baltimore, MD USA; 3 Department of Pharmaceutical Sciences, University of Maryland School of Pharmacy, Baltimore, MD USA

## Abstract

**Background:**

SARS-CoV-2 causes COVID-19 which has a widely diverse disease profile. The mechanisms underlying its pathogenicity remain unclear. We set out to identify the SARS-CoV-2 pathogenic proteins that through host interactions cause the cellular damages underlying COVID-19 symptomatology.

**Methods:**

We examined each of the individual SARS-CoV-2 proteins for their cytotoxicity in HEK 293 T cells and their subcellular localization in COS-7 cells. We also used Mass-Spec Affinity purification to identify the host proteins interacting with SARS-CoV-2 Orf6 protein and tested a drug that could inhibit a specific Orf6 and host protein interaction.

**Results:**

We found that Orf6, Nsp6 and Orf7a induced the highest toxicity when over-expressed in human 293 T cells. All three proteins showed membrane localization in COS-7 cells. We focused on Orf6, which was most cytotoxic and localized to the endoplasmic reticulum, autophagosome and lysosomal membranes. Proteomics revealed Orf6 interacts with nucleopore proteins (RAE1, XPO1, RANBP2 and nucleoporins). Treatment with Selinexor, an FDA-approved inhibitor for XPO1, attenuated Orf6-induced cellular toxicity in human 293 T cells.

**Conclusions:**

Our study revealed Orf6 as a highly pathogenic protein from the SARS-CoV-2 genome, identified its key host interacting proteins, and Selinexor as a drug candidate for directly targeting Orf6 host protein interaction that leads to cytotoxicity.

**Supplementary Information:**

The online version contains supplementary material available at 10.1186/s13578-021-00568-7.

## Introduction

SARS-CoV-2 (severe acute respiratory syndrome coronavirus 2) is a novel coronavirus responsible for causing the coronavirus disease 2019 (COVID-19) pandemic [4, 13]. To date, SARS-CoV-2 has infected more than 78 million people around the world and led to over 1,7 million deaths (source: Johns Hopkins University). This is the third introduction of a highly pathogenic coronavirus into the human population in the twenty-first century, following SARS-CoV (2002–2003) and MERS-CoV (Middle East respiratory syndrome-CoV; 2012). Many of the studies on SARS-CoV-2 and ongoing drug development have been focused on virus entry and replication. However, functions of individual SARS-CoV-2 viral proteins, particularly how they affect human cells, remain largely unknown.

The SARS-CoV-2 viral genome encodes 28 confirmed proteins. The 5′ of the viral genome contains Orf1ab, by far the largest viral gene, encoding polyproteins Orf1ab and Orf1a. These polyproteins are cleaved into 16 non-structural proteins (Nsp1-16) that form the viral transcription/replication complex, such as papain-like proteinase (PL^pro^; Nsp3) [18], RNA-dependent RNA polymerase (RdRp,Nsp12 in complex with Nsp7-Nsp8 heterodimer co-factor) [20], nucleoside triphosphate hydrolase (NTPase) and helicase (Nsp13) [36], exonuclease (Nsp14) [1], endoribonuclease (NendoU,Nsp15) [17], and RNA 5′ cap structure (Nsp14 combined with the Nsp16-Nsp10 methyltransferase complex) [41]. The remaining viral genome sequence at the 3′ end comprises genes encoding the four structural proteins spike (S), envelope (E), membrane (M) and nucleocapsid (N), as well as eight accessory proteins (Orf3a, Orf3b, Orf6, Orf7a, Orf7b, Orf8, Orf9b and Orf10) [12, 47]. The functions of these accessory proteins remain largely unresolved since they lack well-defined domain structures. Studies of the previous SARS-CoV and MERS-CoV coronaviruses have hinted that these accessory proteins might interact extensively with host proteins to facilitate viral growth and replication [26, 27].

The limited viral genome compels the virus to enlist host systems for its cellular infection, translation, replication and spread. Understanding how SARS-CoV-2 hijacks host machinery and which of its proteins are key for its interaction will be crucial in identifying effective targets for COVID-19 therapeutic intervention. Studies of the human immune deficiency virus (HIV-1) over the past 30 years have demonstrated the presence of prime pathogenic proteins that contribute to virulence and host disease progression, and the potential of targeting these proteins for effective therapeutic intervention [9]. For example, HIV-1 Vpr protein interacts with host proteins to modify host cell energy metabolism, oxidative status and proteasome function. This protein’s effect on critical host systems contributes to disease severity and makes Vpr a primary determinant of HIV-1 pathogenesis. As such, Vpr, as well as its main host interacting proteins, are currently studied as viable potential pharmacological targets for treating HIV-associated diseases [11]. The HIV-1 Tat protein (another prime pathogenic HIV-1 protein) activates HIV-1 gene expression through binding host proteins such as TAK, CDK9 and Cyclin T1 ([43, 46. Therefore, small molecules that target this interaction are expected to inhibit HIV-1 virus gene expression and are currently being developed [33]. The HIV-1 Nef protein is another important determinant of viral pathogenesis [2]. Nef is abundantly expressed during infection and reroutes a variety of cell surface proteins to disrupt host immunity and promote the viral replication cycle [2]. Nef also counteracts host defenses by sequestering and/or degrading its targets via the endocytic and secretory pathways, through direct interaction with a number of host trafficking proteins [29, 31, 35]). Therefore, inhibiting HIV-1 Nef protein or its interacting host factors provides a promising approach for future drug development [2]. These studies have demonstrate that examining individual viral proteins in cell culture and animal models can provide valuable insights into the underlying disease mechanisms. And, that identification of these prime pathogenic proteins encoded by the virus genome will likely be critical for developing targeted therapeutic treatments that benefit patients.

To identify the key pathogenic proteins of SARS-CoV-2, we expressed each of the confirmed 28 proteins encoded by the SARS-CoV-2 genome in human cells. We then examined which individual proteins affected cell viability and their subcellular localization. Multiple viral genes were found to induce cytotoxicity, among which Orf6, Orf7a and Nsp6 were the most potent, with each showing a particular subcellular localization pattern. Orf6 protein showed a distinct distribution pattern with localization exclusively at the endoplasmic reticulum (ER), autophagosome and lysosome membranes. Since Orf6 also caused the highest cytotoxicity in our assay, we examined it more closely. We used affinity-purification mass spectrometry to identify Orf6 viral-host protein interactions. The data revealed SARS-CoV-2 Orf6 interaction with host proteins associated with RNA transport and localization, ribosome and proteasome complexes and MHC class I antigen processing pathways. The host RNA transport and localization proteins included several nuclear pore proteins (RAE1, XPO1, RANBP2 and multiple nucleoporins). Therefore, we tested two FDA-approved nuclear pore inhibitors. We found that Selinexor, a selective inhibitor of nuclear export [15]—but not Ivermectin, an inhibitor of nuclear import [42]—attenuated SARS-CoV-2 Orf6-induced cellular toxicity in a dose-dependent manner. These findings suggest that Selinexor, and possibly other nuclear transport inhibitors, could be used as a targeted treatment for SARS-CoV-2 Orf6 protein-induced cellular damage through blocking its interaction with nuclear pore proteins.

## Results

### Cellular toxicity of the individual SARS-CoV-2 genes

**Fig. 1:**
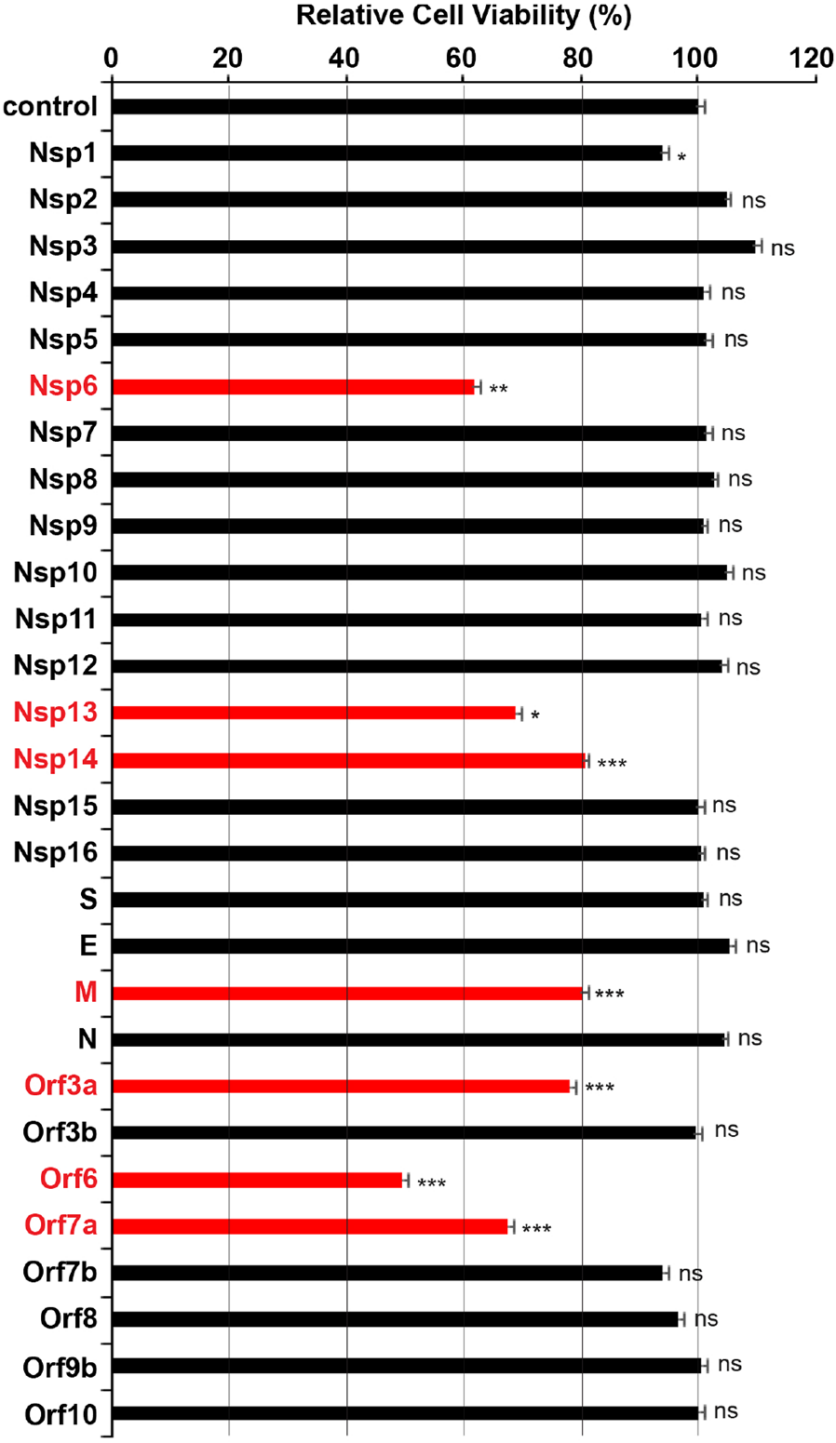
Cellular toxicity of SARS-CoV-2 genes. HEK 293 T cells were transfected with 150 ng of plasmid for each SARS-CoV-2 gene, and cell viability was analyzed by a luminescent cell viability assay. Red color indicates statistically significant cell death induced by viral gene expression (mean ± SD; n = 3 independent experimental replicates; T-test with Bonferroni correction between the control and viral gene expressing cells; *P < 0.05, **P < 0.01, ***P < 0.001; *ns* non-significant)

The SARS-CoV-2 genome contains 28 genes that encode 16 non-structural proteins, 4 structural proteins and 8 accessory proteins. To investigate potential pathogenic effects caused by the 28 individual SARS-CoV-2 genes, we expressed each gene in HEK 293 T cells (each was driven by the same promotor and all transfections were carried out with equal amounts of construct) and analyzed effects on cell viability (Fig. 1). We identified seven SARS-CoV-2 proteins capable of inducing significant cell viability defects (Fig. 1, red bars). Among these seven, SARS-CoV-2 Orf6 displayed the strongest toxicity phenotype with near 50% loss in cell viability, followed by the Nsp6 and Orf7a proteins which each showed ~ 30–40% reduction in cell viability (Fig. 1). Four other proteins (Nsp13, Nsp14, Orf3a and M) induced milder yet significant cell death, whereas the other 21 proteins did not induce detectable cellular toxicity (Fig. 1).

### Intracellular localization of the individual SARS-CoV-2 proteins

Knowledge of the subcellular localization pattern of individual SARS-CoV-2 proteins could aid to infer their biological function based on the host proteins and processes available at the cellular compartment. The subcellular localization of a protein can be predicted based on the presence of topological motifs such as a transmembrane (TM) domain, signal peptide, membrane-embedded alpha-helix or nuclear localization sequence. Therefore, we summarized the predicted topology and subcellular localization for the 28 SARS-CoV-2 proteins (Additional file 1: Table S1). We used these predictions, combined with data from UniProt Knowledgebase [40] and the scientific literature, to prioritize the SARS-CoV-2 proteins as a means to accelerate our initial round of investigation. Viral proteins with main predicted functions in viral entry, replication or packaging were initially not favored as they remain a focus of much of the latest literature. Twelve SARS-CoV-2 proteins (Nsp1, Nsp2, Nsp3, Nsp6, Orf3a, Orf3b, Orf6, Orf7a, Orf7b, Orf8, Orf9b and Orf10) were thus prioritized for analysis of their subcellular localization by super-resolution confocal microscopy. We expressed the mCherry (mCh)-fused (C-terminus) SARS-CoV-2 proteins in COS-7 cells along with a fluorescent ER marker, mCitrine (mCi)-ER (Note, when highly overexpressed this marker is prone to label the Golgi structure as well). COS-7 cells are a mammalian cell line commonly used for protein subcellular localization studies due to their large size. We chose mCi-ER because the ER is an abundant cellular structure ideal for observing subcellular distribution of proteins, as well as a major organelle at which many viral proteins tend to localize (Additional file 1: Fig. S1).

**Fig. 2:**
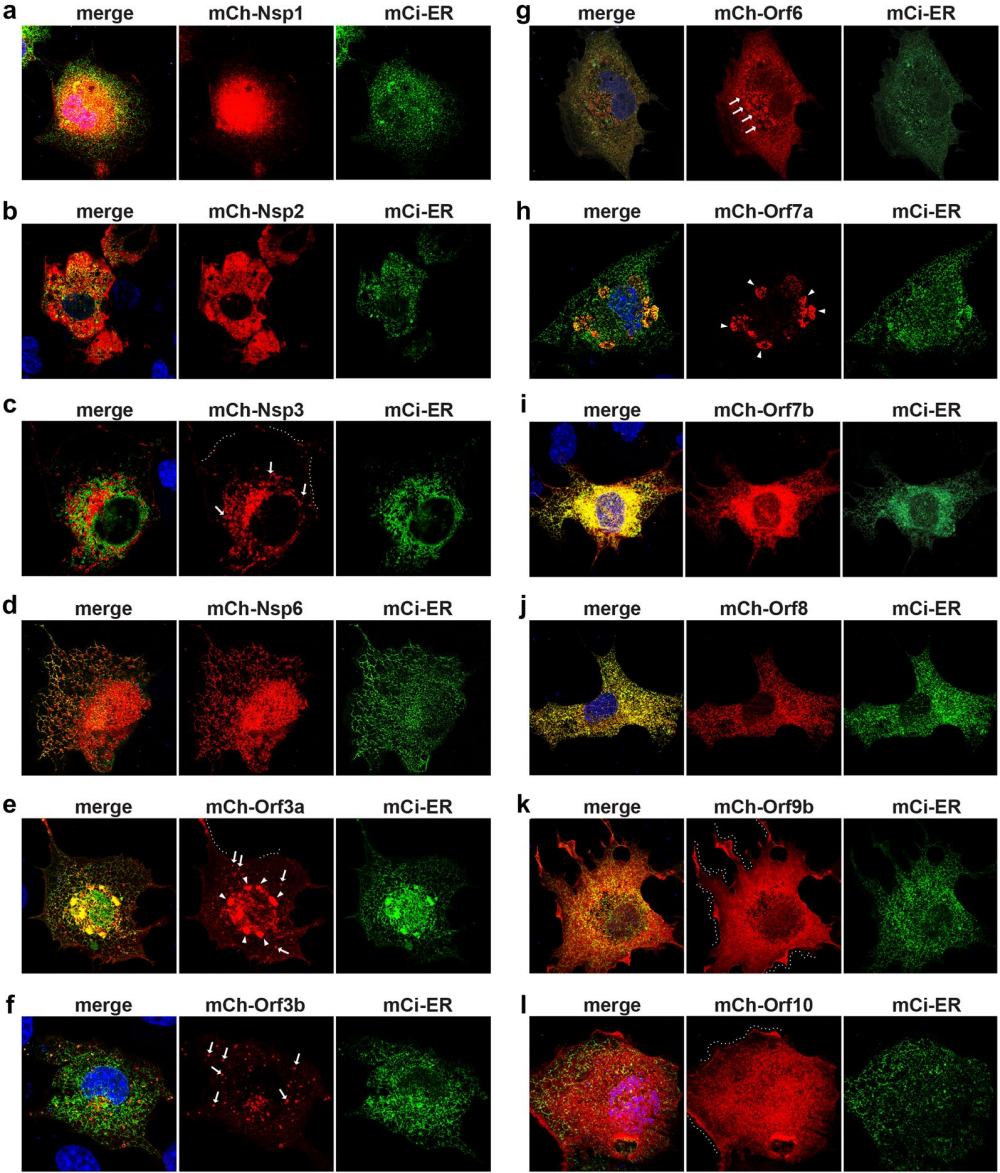
Cellular localization of SARS-CoV-2 proteins. COS-7 cells co-transfected with mCherry (mCh)-tagged SARS-CoV-2 proteins (**a** Nsp1, **b** Nsp2, **c** Nsp3, **d** Nsp6, **e** Orf3a, **f** Orf3b, **g** Orf6, **h** Orf7a, **i** Orf7b, **j** Orf8, **k** Orf9b, **l** Orf10) and the monomeric Citrine (mCi)-tagged ER marker were imaged by confocal microscopy. Dotted line follows plasma membrane; arrow points to intracellular vesicle; arrowhead indicates Golgi structure

Nsp1 is localized in the cytosol and nuclear region, although it does not contain a known-nuclear localization sequence (Fig. 2a). Nsp2 shows typical cytosolic distribution (Fig. 2b). Nsp3 has two TM domains and localizes at the membranes of vesicles (Fig. 2c, arrows) and the plasma membrane, but not the ER (Fig. 2c). Nsp6 has six TM domains and shows ER and perinuclear localization (Fig. 2d). Orf3a contains three known TM domains, it localizes mostly at the Golgi (Fig. 2e, arrowheads) and at the lumen of particular vesicles (Fig. 2e, arrows), and only weakly at the ER and plasma membrane (Fig. 2e). Orf3b shows weak cytosolic signal and strong vesicular luminal signal (Fig. 2f, arrows). Orf6 has a membrane-embedded alpha-helix on its N-terminus and localizes to the ER and the membranes of a subset of the intracellular vesicles (Fig. 2g, arrows). Orf7a has a signal peptide on its N-terminus and one TM domain. It localizes predominantly at the Golgi (Fig. 2h, arrowheads) and shows a weak ER signal (Fig. 2h). We observed that Orf7a also localizes at mitochondria in a small subset of cells (data not shown). Orf7b and Orf8 co-localize almost completely with the mCi-ER marker (Fig. 2i, j). Orf9b and Orf10 show cytosolic distribution and plasma membrane localization (Fig. 2k, l). In addition, Orf10 shows nuclear localization (Fig. 2l). These observations have been summarized in Table 1.

**Table 1:** The summary of cellular localization of SARS-CoV-2 proteins

SARS-CoV-2 Protein	Confirmed subcellular localization by confocal microscopy
Nsp1	cytosol, perinuclear region
Nsp2	cytosol
Nsp3	membrane of vesicles, plasma membrane, not ER
Nsp6	ER, perinuclear region
Orf3a	Golgi, weakly at ER, plasma membrane, lumen of vesicles
Orf3b	cytosol, lumen of vesicles
Orf6	ER, membrane of vesicles (autophagosome, lysosome)
Orf7a	mostly at Golgi, weekly at ER and mitochondria
Orf7b	ER
Orf8	ER
Orf9b	cytosol, plasma membrane
Orf10	cytosol, plasma membrane, perinuclear region

### SARS-CoV-2 Orf6 localizes to the endoplasmic reticulum, autophagosome and lysosomal membranes

**Fig. 3:**
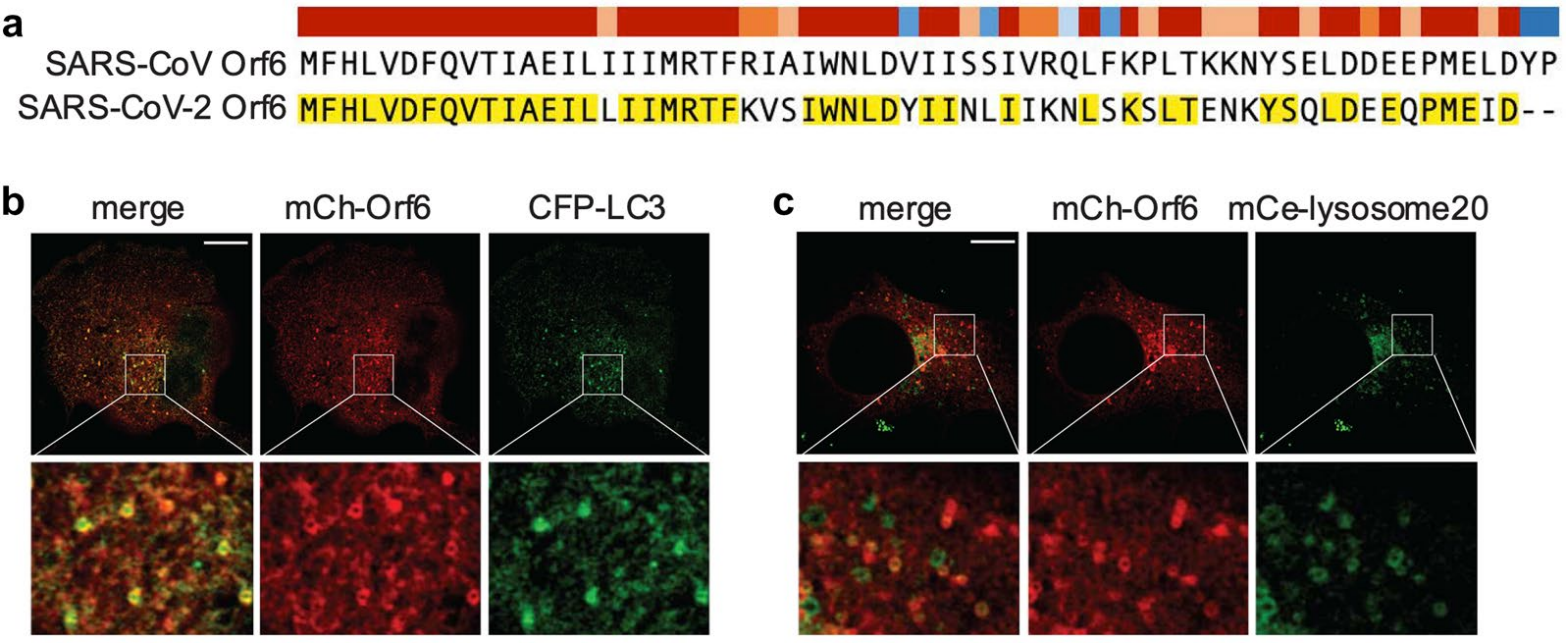
SARS-CoV-2 Orf6 is localized at the ER, autophagosome and lysosome membranes. **a** Schematic homology comparison between SARS-CoV Orf6 and SARS-CoV-2 Orf6. **b**, **c** Confocal analysis of SARS-CoV-2 protein Orf6 localization in COS-7 cells transfected with mCh-Orf6 together with CFP-LC3 (**b**), and mCe-lysosome20 (**c**). Scale bar, 10 μm. CFP, cyan fluorescent protein; LC3, microtubule-associated protein 1A/B-light chain 3; mCe, mCerulean; mCh, mCherry

SARS-CoV-2 Orf6 protein showed the most potent cellular toxicity and displayed an intriguing ER and vesicular localization pattern, which led us to investigate this virus protein further. It has been reported that SARS-CoV Orf6 is localized on the ER and Golgi membranes due to the alpha-helix on its N-terminus [10]. This N-terminal alpha-helix is conserved in SARS-CoV-2 Orf6 (Fig. 3a). Interestingly, we found that SARS-CoV-2 Orf6 protein localized at the membrane of particular vesicles as well as the ER (Fig. 2g). To better characterize the types of vesicles that showed SARS-CoV-2 Orf6 protein localization, we co-expressed Orf6-mCh with additional cellular vesicle markers, including CFP-LC3 (microtubule-associated protein 1A/B-light chain 3) to label autophagosomes, mCerulean (mCe)-lysosome20 to label lysosomes, and endosome vesicle markers. Strikingly, we found that SARS-CoV-2 Orf6 overlaps highly with CFP-LC3 labeled autophagosomes (Fig. 3b), and partially overlapped with mCe-lysosome20 labeled lysosomes (Fig. 3c). However, we found no co-localization with markers for the early or late endosomes (mCi-Rab5a, mCi-Rab7a, mCi-Rab9a,Additional file 1: Fig. S2). Although the N-terminus alpha-helix has been conserved, SARS-CoV-2 and SARS-CoV Orf6 only show about 67% overall similarity in codon sequence [12]. We speculate this low sequence conservation might result in differences in host protein interaction and the associated viral regulation of host systems.

### SARS-CoV-2 Orf6 virus-host protein interactions

**Fig. 4:**
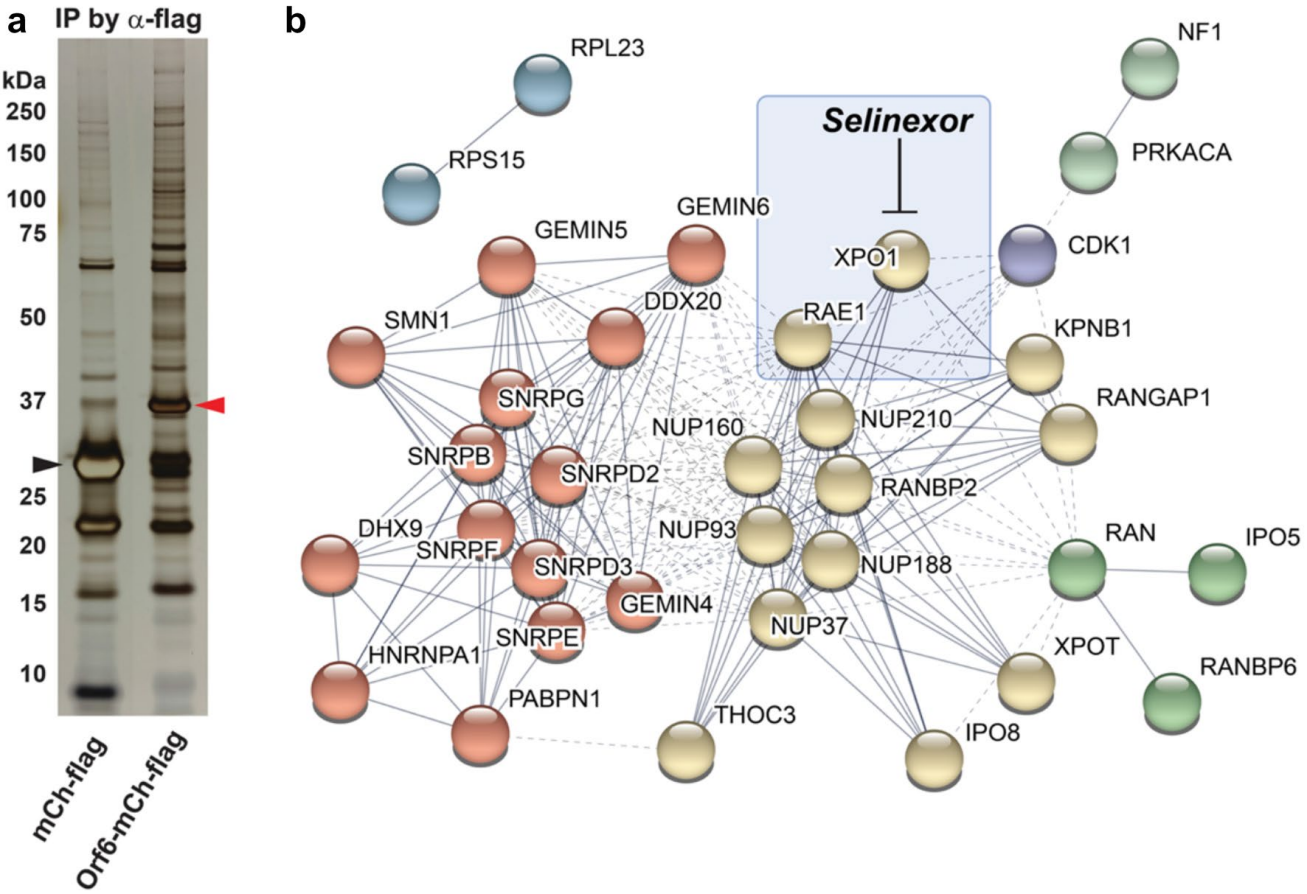
Host-interacting proteins of SARS-CoV-2 Orf6. **a** Cells transfected with either a control (mCherry (mCh)-FLAG) or Orf6-mCh-FLAG-expression vector were lysed. Conjugated-proteins were pulled-down using FLAG agarose beads. The pulled-down samples were analyzed by SDS-PAGE gel and visualized by silver stain (black arrowhead, mCh-FLAG; red arrowhead, Orf6-mCh-FLAG). **b** Graphic network display of the interactions between SARS-CoV-2 Orf6 and host proteins associated with “nuclear transport (GO:0051169)”. Interactions were identified by STRING, then proteins were clustered using Markov Cluster Algorithm and colored based on the result. Solid line, intra-cluster association; dashed line; inter-cluster association

Orf6 proteins from SARS-CoV and SARS-CoV-2, as expected, share functionality, including IFN-antagonistic activity [21, 28, 50] and interaction with the NUP98-RAE1 complex at the nuclear pore [10, 12, 24, 25, 28]. To gain insight into SARS-CoV-2 Orf6 regulated host processes, we carried out affinity purification followed by high resolution mass spectrometry. We expressed mCh-FLAG-tagged Orf6 in HEK 293 T cells and performed co-immunoprecipitation assays using anti-FLAG agarose. The pulled-down proteins were separated by SDS-PAGE gel and visualized by silver stain. Several unique bands were observed in the Orf6-mCh-FLAG sample compared to the control (mCh-FLAG) (Fig. 4a).

Gene Ontology analysis of the Orf6 interacting host proteins identified by mass spectrometry (Additional file 1: Table S2) revealed host functions that are potentially modulated by SARS-CoV-2 Orf6 to support viral replication. For example, we identified host proteins that are required for translational initiation (GO:0006413, adjusted *p* value = 5.79e−32), viral gene expression (GO:0019080, adj. *p* = 7.14e−28), antigen processing and presentation (GO:0042590, adj. *p* = 1.11e−27), and interleukin-1-mediated signaling (GO:0070498, adj. *p* = 4.95e−26), among others. Remarkably, our survey revealed nuclear transport-related proteins (GO:0051169, adj. *p* = 1.57e−15) as a major functional category (Fig. 4b). This finding is in line with previous interactions [12, 24, 25] and recent demonstration [28] that SARS-CoV-2 Orf6 interacts with the host RAE1-NUP98 complex. Our Orf6-nuclear pore interaction network included nucleoporins (RAE1, RANBP2 [as well as RAN and RANGAP1], NUP160, NUP188, NUP210, NUP37, NUP93), importins (IPO5, IPO8, RANBP6, KPNB1) and exportins (XPO1, XPOT [a.k.a. XPO3]). Interestingly, THOC3, a member of the TREX complex and known for coupling mRNA processing with transport, as well as several members of the spliceosome were also present in the interaction network. The latter include small nuclear ribonucleoproteins (SNRPs), a heterogenous nuclear ribonucleoprotein (HNRNPA1), and several Gem-associated protein (GEMIN) family members. Taken together these findings expand on what was known about the host nuclear pore interaction network affected by SARS-CoV-2 Orf6 protein.

### SARS-CoV-2 Orf6 cellular toxicity is attenuated by Selinexor treatment

**Fig. 5:**
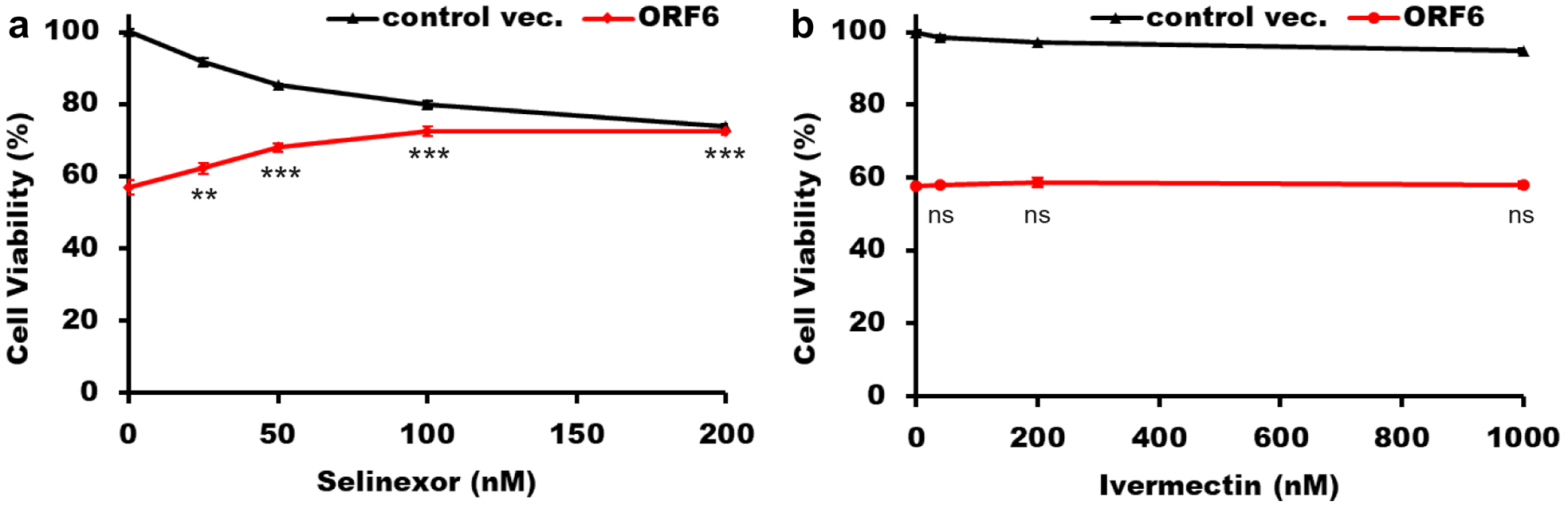
Orf6-induced cellular toxicity is attenuated by treatment with Selinexor. HEK 293 T cells were transfected with 150 ng of control vector or Orf6 expression vector and treated with 0, 25, 50, 100, and 200 nM Selinexor (**a**) or with 0, 40, 200, 1000 nM Ivermectin (**b**). Cell viability was analyzed by luminescent cell viability assay (mean ± SD; n = 3 independent samples; T-test with Bonferroni correction between the non-treated (0 nM) and drug-treated cells; **P < 0.01, ***P < 0.001; *ns* non-significant)

It has been predicted that Orf6 interaction with the mRNA nuclear export complex (RAE1 and NUP98) can be disrupted by Selinexor, an FDA-approved selective inhibitor of nuclear export [12]. To investigate whether Selinexor can diminish the cellular toxicity caused by Orf6, we treated SARS-CoV-2 Orf6 transfected HEK 293 T cells with Selinexor and analyzed cell viability. Even though Selinexor itself appeared toxic to the cells (Fig. 5a, control vec.), its treatment was able to attenuate SARS-CoV-2 Orf6-induced cellular toxicity and it did so in a dose-dependent manner (Fig. 5a). We also tested Ivermectin, another FDA-approved nuclear transport inhibitor, that had been suggested as a putative inhibitor for SARS-CoV-2 Orf6 [3]. However, Ivermectin did not demonstrate capability of reducing SARS-CoV-2 Orf6-induced cellular toxicity (Fig. 5b). These findings suggest that Selinexor, and possible other nuclear transport inhibitors, could be used as a targeted treatment for SARS-CoV-2 Orf6 protein induced cellular damage through blocking its interaction with nuclear pore proteins.

## Discussion

SARS-CoV-2, like all viruses, is dependent on host cell systems for infection, translation, replication and spread. Studies of HIV-1 over the past 30 years have demonstrated the importance of identifying the major contributing pathogenic viral proteins as potential therapeutic targets in drug discovery [9]. In this study, we have used both cytotoxicity and subcellular localization to identify prime pathogenic proteins encoded by SARS-CoV-2. Previous studies of SARS-CoV proteins found that viral Orf3a, Orf3b, Orf7a and Orf7b protein expression induced cytotoxicity [16, 22, 23, 34, 49]. Interestingly, all four are accessory proteins, which show the least conservation of amino acid sequence between the SARS-CoV and the current SARS-CoV-2 [12]. This highlights the importance of studying the SARS-CoV-2 proteins directly rather than relying on deductions based on SARS-CoV data. Our systematic cytotoxicity screen for all 28 SARS-CoV-2 proteins revealed that seven proteins (Orf6, Nsp6, Orf7a, Nsp13, Nsp14, Orf3a and M) were capable of inducing cytotoxicity when expressed in human cells (HEK 293 T) (Fig. 1). Among them, Orf6, Nsp6 and Orf7a were highly cytotoxic, and therefore presumed pathogenic. We found these viral proteins (SARS-CoV-2 Orf6, Nsp6 and Orf7a) were similarly highly toxic in our in vivo* Drosophila* model [52]. Cytotoxicity caused by Orf3a was relatively mild and fell within the range of other cytotoxic proteins with milder effects (Nsp13, Nsp14 and M). A recent study reported a similar level of cytotoxicity for SARS-CoV-2 Orf3a expression in HEK 293 T, Vero E6 and HepG2 cell lines [32]. They found Orf3a induced apoptosis via caspase-3 activation and showed that this effect was stronger for the previous SARS-CoV Orf3a. These data imply that similar to other viruses, SARS-CoV-2 harbors primary determinant pathogenic proteins. We speculate these might hold the key to the tissue-specific effects and symptomatology observed in COVID-19, which likely dependent on specialized virus-host protein interactions. Our report using *Drosophila* to investigate SARS-CoV-2 protein pathogenicity supports this notion by demonstrating the detrimental effects of these proteins on multiple tissues. These included mitochondrial damage observed in muscle of Orf6, Nsp6 and Orf7a transgenic flies, which might be a prelude to apoptosis [52].

Knowledge of the subcellular localization pattern of individual SARS-CoV-2 proteins could aid interpretation of their biological functions based on the host proteins and systems available for interaction within the cellular compartment. Overall, when comparing our data with that published in the literature for SARS-CoV, we found many similarities. However, we also uncovered notable differences in viral protein localization between SARS-CoV-2 and SARS-CoV (see, Additional file 1: Discussion), indicating the importance of studying SARS-CoV-2 directly. Strikingly, examination of viral protein localization in COS-7 cells revealed that the three SARS-CoV-2 proteins (Orf6, Nsp6 and Orf7a) which displayed the highest potency for cytotoxicity, are localized on the membranes of the ER, Golgi system and a subset of intracellular vesicles. Orf7a showed a large presence at the Golgi compartment, where it might play a role in mediating the host cell trafficking process. It has been reported that SARS-CoV Orf6 is required for optimal replication of the SARS-CoV virus and is localized at the ER and Golgi membranes [10]. Although the protein sequence of SARS-CoV-2 Orf6 has only ~ 67% similarity to the codon sequence of SARS-CoV Orf6, the N-terminus alpha-helix motif is conserved. This alpha-helix is required for optimal replication of the SARS-CoV virus and important for localization of the SARS-CoV Orf6 protein to the ER and Golgi membranes [51]. We observed that SARS-CoV-2 Orf6 localized to the ER and the membranes of intracellular vesicles. Previous technology did not allow for the level of high-resolution images we were able to achieve in our study, which could explain the absence of intracellular vesicles in the previous observation. Alternatively, these Orf6-marked vesicles might be unique to SARS-CoV-2 Orf6. Combined with the low level of conservation of protein sequence, these could indicate that while certain important functions of SARS-CoV Orf6, such as enhancing viral replication [51] and viral growth [30] might be conserved in SARS-CoV-2 Orf6, the latter displays additional features associated with altered host protein interaction. Interestingly, further investigation of SARS-CoV-2 Orf6 protein localization showed it was specifically present at autophagosomes and lysosomes, but not at early or late endosomes. This pattern, including ER localization, suggests that SARS-CoV-2 Orf6 protein might interact with the host protein trafficking, autophagy and protein degradation.

SARS-CoV-2 Orf6 has been shown to act as an antagonist of IFN signaling [24, 25, 28, 50] as well as to directly interact with the NUP98-RAE1 complex at the nuclear pore [12, 24, 25, 28]. To gain insight into SARS-CoV-2 Orf6 regulated host processes, we carried out affinity purification followed by high resolution mass spectrometry. In line with previous findings, we found SARS-CoV-2 Orf6 protein interacts with many key members of the nuclear pore machinery. Our data identified nucleoporins and karyopherins (both importins and exportins), as well as components of the spliceosome among the interactors. Among them we identified RAE1, XPO1, RANBP2 and additional components of Ran-GTP karyopherin-mediated protein transport and nuclear pore machinery, but notably not NUP98. At the time of finalizing this manuscript, a study reported that interaction of SARS-CoV-2 Orf6 by itself with the NUP98-RAE1 complex is sufficient in suppressing IFN signaling [28]. Evading the innate immune response is imminent to virus survival and targeting host transport systems is a common tactic, however the mechanisms of intervention varies among viruses. SARS-CoV-2, and its predecessor SARS-CoV, target the nuclear import of STAT1 and STAT2 to impede the induction of IFN-stimulated genes [28]. The karyopherin alpha1 (KPNA1) and beta 1 (KPNB1) heterodimer is key to importing the STAT signaling complex into the nucleus. Interestingly, we found KPNB1 among the SARS-CoV-2 Orf6 interactors (Fig. 4b). Its binding site is closely located to that of RAE1 on NUP98 and might also be disrupted by Orf6 binding. Indeed, the latest report showed Orf6 directly binds NUP98 and suggested in doing so it might disrupt a wider range of transport than IFN signaling alone [28]. Interestingly, in addition to nuclear importers, we detected export components in our Orf6 interaction network (Fig. 4b). Among these, THOC3 is part of a nuclear complex involved in transcription elongation (THO), which in turn is part of the larger transcription export (TREX) complex. TREX is involved in splicing-coupled mRNA export [19]. Components of the spliceosome comprised a sizeable part of the proteins closely interacting with the nuclear pore in our SARS-CoV-2 Orf6 virus-host interaction network. Together these findings indicate that in addition to the previously reported IFN immune suppression through disrupted nuclear import [28, 50], Orf6 might hamper host RNA export mechanisms. These could further suppress the immune response and free up host translation machinery for processing viral RNA to ramp up viral replication. This is supported by findings that total RNA content is dramatically reduced in SARS-CoV-2 infected cells [28]. Our findings identified additional proteins involved in this process, including components of spliceosome-mediate transport, and expand on what was known about SARS-CoV-2 Orf6 disruption of host nuclear pore function.

**Fig. 6:**
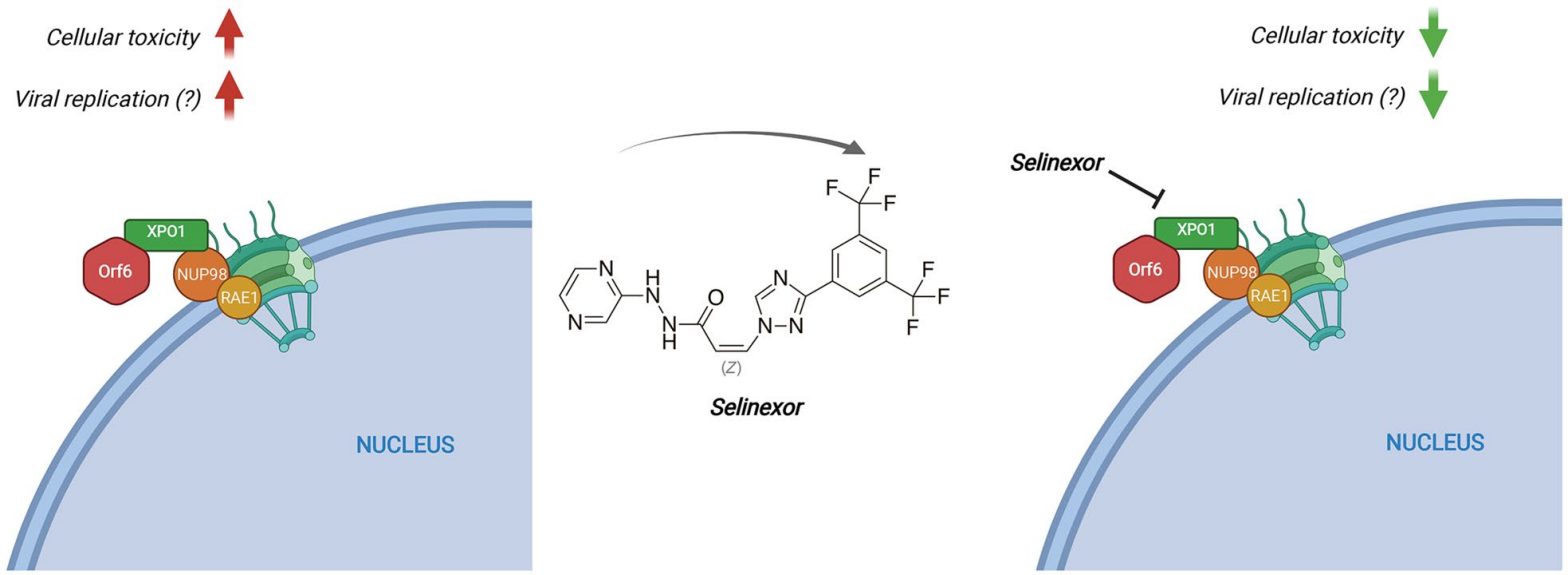
Model for SARS-CoV-2 Orf6 pathogenic mechanism. Panel on the left shows SARS-CoV-2 Orf6 binds XPO1 at the host nuclear pore complex, which leads to the observed cellular toxicity. Viral control of host nuclear transport is a common virus tactic to evade the host immune response and could potentially pave the way for increased viral replication. The right panel illustrates Selinexor’s mode of action, by directly binding and inhibiting XPO1 activity it prevents SARS-CoV-2 Orf6 from hijacking the host nuclear pore complex and thus inhibits Orf6 cellular toxicity

Identification of primary determinant pathogenic proteins for HIV-1, such as Vpr, Tat and Nef, has provided novel therapeutic targets for treating HIV-1-associated diseases [9]. Therefore, we sought to identify potential drugs that could target SARS-CoV-2 Orf6, or its interacting host proteins, as potential therapeutics for COVID-19. Fortunately, XPO1, one of the prime host interaction proteins for Orf6 identified in our data, is the direct target of Selinexor (Fig. 6). Selinexor is an FDA approved-selective inhibitor of nuclear export [38]. The UCSF group predicted Selinexor among one of the 69 drugs that could inhibit SARS-CoV-2 and human protein interactions, based on the indirect association with RAE1 [12] which our study and others [24, 25] picked up as well. RAE1 is another key component of the nuclear export complex machinery and contains a specific binding pocket for XPO1. We tested Selinexor treatment directly in our HEK 293 T cell model and found that Selinexor treatment effectively attenuated the cellular toxicity caused by Orf6 expression, in a dose-dependent manner. We also tested Ivermectin, another nuclear transport inhibitor, that has been suggested as a putative inhibitor for SARS-CoV-2 Orf6 [3]. However, Ivermectin was not able to detectably reduce SARS-CoV-2 Orf6-induced cytotoxicity. Selinexor directly targets XPO1, the nuclear export host protein, thereby inhibiting SARS-CoV-2 Orf6 viral-host protein interaction [15] (Fig. 6), whereas Ivermectin indirectly inhibits nuclear import via the importin alpha/beta-mediated pathway [42]. Furthermore, we report therapeutic intervention by Selinexor is similarly effective in vivo, wherein we showed Selinexor treatment attenuated SARS-CoV-2 induced pathogenic effects in fly trachea (*Drosophila* equivalent of lung) and muscle tissue [52].

Currently pharmaceutical targets for treating COVID-19 focus on inhibition of either virus entry (via blocking Spike-ACE2 binding or inhibition of TMPRSS2 activity; for example, camostat mesylate) or virus replication (via inhibition of the virus RNA-dependent RNA polymerase (RdRp); for example, Remdesivir). We speculate that pharmaceutical compounds that target the specific primary determinants of SARS-CoV-2 pathogenicity, as we demonstrated here for Orf6, will be most beneficial in combination therapy with the viral entry/replication inhibitors and will act to mitigate damage to the multiple tissues affected in COVID-19.

## Methods

### Cell lines, plasmids and transfection

The HEK 293 T and COS-7 cells were purchased from ATCC and maintained in DMEM medium (Corning cellgro) containing 10% fetal bovine serum and Penicillin–Streptomycin (10 unit/ml). Mammalian expression constructs for *Nsp1*, *Nsp2*, *Nsp3*, *Nsp6*, *Orf3a*, *Orf3b*, *Orf6*, *Orf7a*, *Orf7b*, *Orf8*, *Orf9b*, and *Orf10* were generated by using NEBuilder HiFi DNA Assembly Cloning Kit (New England BioLabs). Orf9c was excluded as its genetic code lies not within the verified SARS-CoV-2 open reading frame. The cDNA fragments were PCR-amplified from human codon-optimized SARS-CoV-2 genes in pDONR207 vector (from Fritz Roth, through Addgene) or pLVX-EF1alpha-IRES-Puro vector (from Nevan Krogan, through Addgene), and assembled into the pCMV6 vector for C-terminal tagging with mCherry (mCh)-FLAG. The mCh-tag was used for tracking the subcellular localization of the viral protein, whereas the FLAG-tag was used in the affinity purification/pull-down assays. The mammalian expression constructs for *Nsp4*, *Nsp5*, *Nsp7*, *Nsp8*, *Nsp9*, *Nsp10*, *Nsp11*, *Nsp12*, *Nsp13*, *Nsp14*, *Nsp15*, *Nsp16*, *spike* (*S*), *envelope* (*E*), *membrane* (*M*) and *nucleocapsid* (*N*) were generously gifted by Dr. Xuefeng Liu at Georgetown University. mCitrine-ER-5, mCerulean3-Lysosomes-20, mCitrine-Rab5a-7 and mCitrine-Rab7a-7 were gifts from Michael Davidson (Addgene plasmid # 56557, # 55428, # 56566, and # 56567 respectively). mCitrine-Rab9a was a gift from Yihong Ye (Addgene plasmid # 78596). pEX-CFP-hLC3WT was a gift from Isei Tanida (Addgene plasmid # 24985). Transfection was performed with TransIT-293 (Mirus) for HEK 293 T cells, and with lipofectamine3000 (Invitrogen) for COS-7 cells following the manufacturer’s instructions.

### Cell viability assay

HEK 293 T cells were seeded 1 × 10^4^ cells/well in opaque-walled white 96-well plate. After 24 h of incubation, 150 ng control plasmid (mCh-FLAG) or one of the SARS-CoV-2 gene expression plasmids was transfected into the cells. Following 48 h expression at 37 °C, cell viability was determined using a CellTiter-Glo 2.0 Assay (Promega). Briefly, a volume of CellTiter-Glo reagent equal to the volume of cell culture medium present in each well was added and mixed for 30 min at room temperature on an orbital shaker. Then, the luminescent signal was detected by the Spark Multimode Microplate Reader (Tecan) with 10 ms integration time, using the SparkControl application.

### Treatment with Selinexor or Ivermectin

Selinexor (Selleck, # KPT-330) or Ivermectin (Sigma) was dissolved in dimethyl sulfoxide (DMSO; Sigma) and added to the cell culture medium at indicated concentrations at 4 h post-transfection. Following 48 h expression at 37 °C, cell viability was determined using a CellTiter-Glo 2.0 Assay (Promega; details above).

### Confocal microscopy

To observe the localization of SARS-CoV-2 proteins, COS-7 cells were seeded at 1.5 × 10^5^ per well in a 35 mm dish (ibidi GmbH, Germany) coated with fibronectin. A total of 1 µg plasmid, 0.5 µg SARS-CoV-2 gene plasmid with 0.5 µg cellular marker expression plasmid, were co-transfected into cells using Lipofectamine3000 following the manufacturer’s instructions. Airyscan confocal microscopy (ZEISS) with a 100 nm resolution was performed 20 h post-transfection. Cells were washed with 1XPBS and then fixed with 4% paraformaldehyde. Fixed cells were washed and mounted using Fluoromount-G Mounting Medium, with DAPI nuclear labeling (Thermo Fisher Scientific). Cells were imaged on an LSM 900 confocal microscope equipped with an Airyscan detector array with ZEN operating software (blue edition v3.1; ZEISS).

### Co-immunoprecipitation assay

To pull down SARS-CoV-2 Orf6 interacting proteins, HEK 293 T cells grown in 10 cm culture dishes were transfected with Orf6-mCh-FLAG expression plasmid. Cells were propagated for 48 h prior to lysis in a buffer containing 0.5% NP40, 50 mM Tris–HCl, pH 7.4, 150 mM sodium chloride, 2 mM magnesium chloride, and a protease inhibitor cocktail. The cleared cell extract was incubated with FLAG agarose beads (Sigma) and the bound materials were extensively washed with a buffer containing 0.05% NP40, 50 mM Tris–HCl, pH 7.4, 150 mM sodium chloride, 2 mM magnesium chloride. The pulled-down proteins were eluted using 1 mg/ml FLAG peptide (Sigma) in 0.05% NP40, 50 mM Tris–HCl, pH 7.4, 150 mM sodium chloride, 2 mM magnesium chloride.

### Mass spectrometry assay

The pull-down proteins of Orf6 were solubilized in 5% sodium deoxycholate and were washed, reduced, alkylated and trypsinolyzed in filter [8, 45]. Tryptic peptides were separated on a nanoACQUITY UPLC analytical column (BEH130 C18, 1.7 μm, 75 μm × 200 mm, Waters) over a 165 min linear acetonitrile gradient (3–40%) with 0.1% formic acid on a Waters nano-ACQUITY UPLC system and analyzed on a coupled Thermo Scientific Orbitrap Fusion Lumos Tribrid mass spectrometer [44]. Full scans were acquired at a resolution of 240,000, and precursors were selected for fragmentation by collision-induced dissociation (normalized collision energy at 35%) for a maximum 3 s cycle. Tandem mass spectra were searched against UniProt reference proteomes of *Homo sapiens* and SARS-CoV-2 and the mCh sequence using Sequest algorithm [6] and MS Amanda algorithm [5] with a maximum precursor mass error tolerance of 10 ppm. Carbamidomethylation of cysteine and deamidation of asparagine and glutamine were treated as static and dynamic modifications, respectively. Resulting hits were validated at a maximum false discovery rate of 0.01 using a semi-supervised machine learning algorithm Percolator [14]. Label-free quantification was performed using Minora, an aligned AMRT (Accurate Mass and Retention Time) cluster quantification algorithm (Thermo Scientific, 2017) using extracted ion chromatogram. Protein abundance was measured by a Hi3 method [37]. Quantitation between samples was normalized by mCh abundance.

### Bioinformatic analysis of proteomic data

Keratin proteins were discarded from our mass spectrometry result, as they are a common contaminant. Further, we removed proteins that were detected in the control (mCh) from the Orf6 binding protein list. Then, we performed the Gene Ontology (GO) analysis for the remaining Orf6-interacting proteins, using the R package "clusterProfiler" version 3.14.3 for the analysis [48]. We tested GO term enrichment with a hypergeometric test, corrected with the Holm-Bonferroni method. We generated a list of nuclear transport-related proteins based on the gene association with GO term “nuclear transport (GO:0051169)”. We used STRING database version 11 [39] to identify known interactions among the proteins in the list. The graphic only displays high-confidence interactions (confidence score > 0.7) with evidence based on the experiments or database. The results were clustered using Markov Cluster Algorithm [7], embedded in the STRING database.

## Supplementary Information

**Additional file 1.** Additional tables, figures and discussion.

## Data Availability

The datasets generated and analyzed during the current study are deposited to a public available server provided by the Department of Pharmaceutical Sciences, University of Maryland School of Pharmacy, at the UMB-SOP-MetalloCloud repository with the following link: https://bit.ly/31XL2Sx. All data and materials generated in this study are available publicly upon request.
