# Supplementary material for: Characterization of SARS-CoV-2 proteins reveals Orf6 pathogenicity, subcellular localization, host interactions and attenuation by Selinexor
Source: Cell Biosci. 2021 Mar 25;11:58. doi: 10.1186/s13578-021-00568-7 (PMC7993076; doi:10.1186/s13578-021-00568-7)
Supplement: Supplementary file 1 — Additional file 1. Additional tables, figures and discussion. [file 13578_2021_568_MOESM1_ESM.pdf]

MANUSCRIPT: Lee et al. SARS-CoV-2 protein cytotoxicity, Orf6 host interaction

SUPPLEMENTAL MATERIAL

Supplemental Figures:

Figure S1. Cellular distribution of mCi-ER alone in COS7 cells. The images of mCi-ER (green) with DAPI (blue) and mCi-ER alone are provided here as a control for Figure 2.

Figure S2. Orf6 is not localized on the early- or late- endosomes. COS-7 cells co-transfected with Orf6-mCh and the indicated mCi-tagged Rab proteins were imaged by confocal microscopy: A. Orf6-mCh with mCi-Rab5a (early endosome); B. Orf6-mCh with mCi-Rab7a (late endosome); and, C. Orf6-mCh with mCi-Rab9a (late endosome).

Supplemental Tables:

Table S1. Predicted topology and subcellular localization of SARS-CoV-2 proteins. In brackets, amino acid location of topological domain. C-term, C-terminus; ER, endoplasmic reticulum; Nsp, non-structural protein; N-term, N-terminus; Orf, accessory protein; TM, transmembrane.

Table S2. Gene Ontologies based on host-interacting proteins of Orf6.
A table with the Gene Ontologies listed in Figure 4, and the host proteins associated with each ontology.

Figure S1

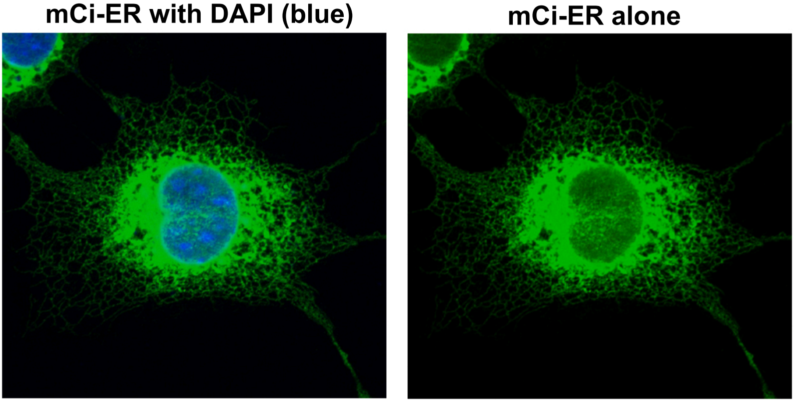

Figure S2.

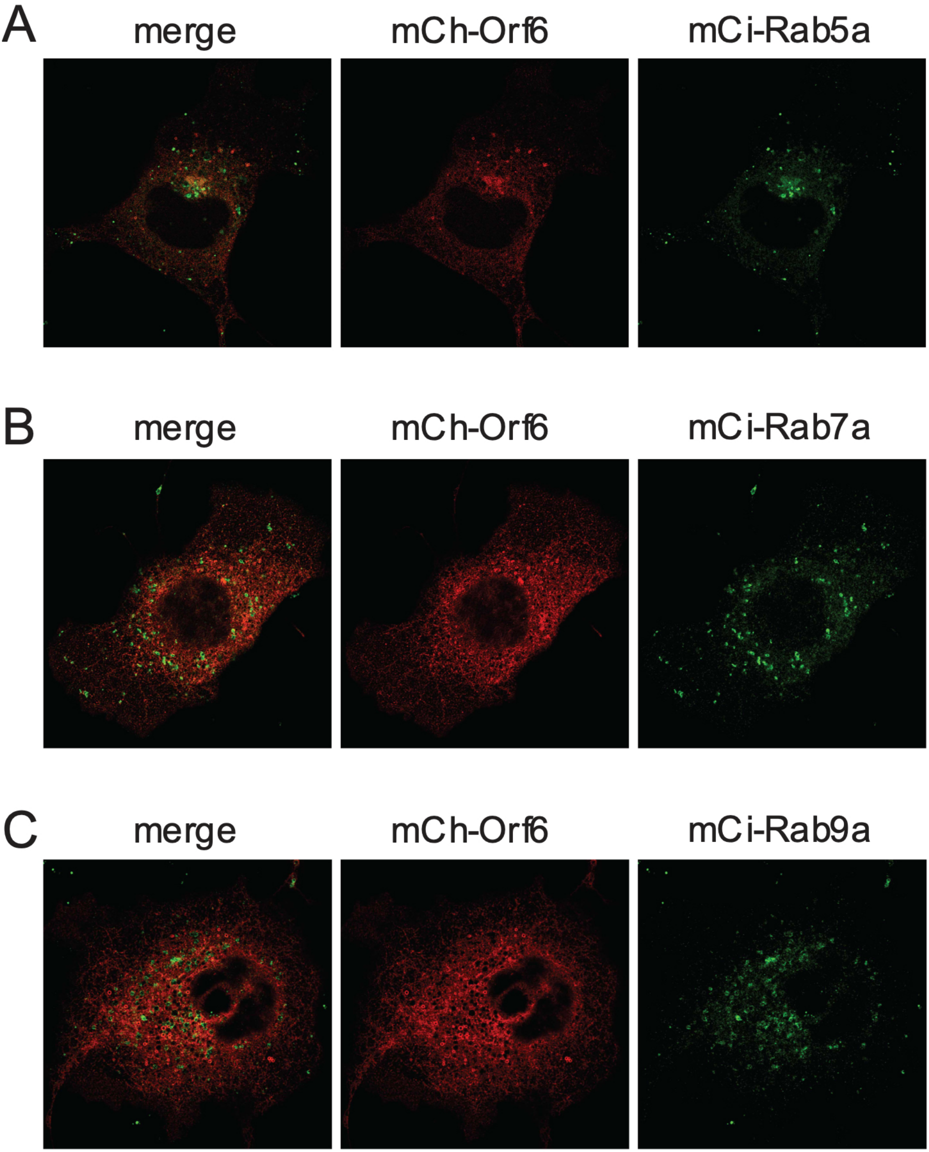
A
merge
mCh-Orf6
mCi-Rab5a
B
merge
mCh-Orf6
mCi-Rab7a
C
merge
mCh-Orf6
mCi-Rab9a

Supplemental Table S1. Predicted topology and subcellular localization of SARS-CoV-2 proteins.

SARS-CoV-2 genes	# of amino acids	Topological domains	Predicted topology and subcellular localization
**Nsp1**	**180**	N/A	cytosol
**Nsp2**	**638**	N/A	cytosol
**Nsp3**	**1945**	2 TM domains	cytoplasmic N-term (1-1047) & cytoplasmic C-term (1521-1945) on membrane
**Nsp4**	**500**	4 TM domains	cytoplasmic N-term (1-13) & cytoplasmic C-term (386-500) on membrane
**Nsp5**	**306**	N/A	cytosol
**Nsp6**	**290**	6 TM domains	cytoplasmic N-term (1-17) & cytoplasmic C-term (231-290) on membrane
**Nsp7**	**83**	N/A	cytosol
**Nsp8**	**198**	N/A	cytosol
**Nsp9**	**113**	N/A	cytosol
**Nsp10**	**139**	N/A	cytosol
**Nsp11**	**13**	N/A	cytosol
**Nsp12**	**932**	N/A	cytosol
**Nsp13**	**601**	N/A	cytosol
**Nsp14**	**527**	N/A	cytosol
**Nsp15**	**346**	N/A	cytosol
**Nsp16**	**298**	N/A	cytosol
**S**	**1273**	signal peptide, 1 TM domains	luminal (13-1213) N-term & cytoplasmic C-term (1235-1273) on membrane
**E**	**75**	1 TM domains	luminal (1-16) N-term & cytoplasmic C-term (38-75) on membrane
**M**	**222**	3 TM domains	luminal (2-19) N-term & cytoplasmic C-term (101-222) on membrane
**N**	**419**	N/A	cytosol
**Orf3a**	**275**	3 TM domains	cytoplasmic N-term (1-34) & luminal C-term (115-275) on membrane
**Orf3b**	**57**	N/A	cytosol
**Orf6**	**61**	membrane-embedded alpha-helix	cytoplasmic C-term on membrane
**Orf7a**	**121**	signal peptide, 1 TM domain	luminal (16-95) N-term & cytoplasmic C-term (117-121) on membrane
**Orf7b**	**43**	1 TM domain	membrane
**Orf8**	**121**	signal peptide	lumen of ER or Golgi
**Orf9b**	**97**	N/A	cytosol
**Orf10**	**38**	N/A	cytosol

MANUSCRIPT: Lee *et al*. SARS-CoV02 protein cytotoxicity, Orf6 host interaction
Supplemental Table S2. Gene Ontologies based n host-interacting proteins of Orf6.

GO ID	Description	Adj. P value	Count	Associated Gene ID
GO:0006402	mRNA catabolic process	1.16634E-44	78	XPO1/PSMD2/PSMD1/PSMC3/PSMC4/PSMC5/PSMC1/PSMC2/PSMD13/PSMC6/PSMD12/TNRC6B/PSMA2/PSMD7/PSMD11/PSMB4/PSMD14/HNRNPU/PSMB1/RPSA/PSMB3/PABPC1/RPL3/PPP2R1A/PSMA5/RPL6/PSMA7/PSMA1/HNRNPM/PSMA4/RPS4X/RPL23/PSMB7/PSMD8/THRAP3/PABPC4/PSMD4/RPS8/RPS18/RPL21/RPS15/PSMA6/PSMA3/ZC3HAV1/RPL5/PSMB2/RPL10/RPS5/RPS3A/DHX9/RPS19/PSME3/RPL8/RPL27/PSMB6/HNRNPA0/RPL15/RPL12/RPL14/RPS13/RPL24/RPL11/TARDBP/RPS28/RPS25/RPS26/RPL10A/VIM/RPS23/RPL36/CNOT1/HNRNPD/RPS9/RPL28/RPL23A/RPL35/RPL30/RPS29
GO:0006401	RNA catabolic process	1.00145E-42	79	XPO1/PSMD2/PSMD1/PSMC3/PSMC4/PSMC5/PSMC1/PSMC2/PSMD13/PSMC6/PSMD12/TNRC6B/PSMA2/PSMD7/PSMD11/PSMB4/PSMD14/LRPPRC/HNRNPU/PSMB1/RPSA/PSMB3/PABPC1/RPL3/PPP2R1A/PSMA5/RPL6/PSMA7/PSMA1/HNRNPM/PSMA4/RPS4X/RPL23/PSMB7/PSMD8/THRAP3/PABPC4/PSMD4/RPS8/RPS18/RPL21/RPS15/PSMA6/PSMA3/ZC3HAV1/RPL5/PSMB2/RPL10/RPS5/RPS3A/DHX9/RPS19/PSME3/RPL8/RPL27/PSMB6/HNRNPA0/RPL15/RPL12/RPL14/RPS13/RPL24/RPL11/TARDBP/RPS28/RPS25/RPS26/RPL10A/VIM/RPS23/RPL36/CNOT1/HNRNPD/RPS9/RPL28/RPL23A/RPL35/RPL30/RPS29
GO:0019080	viral gene expression	4.29198E-33	51	PSMC3/NUP188/NUP160/EIF3B/RANBP2/EIF3A/NUP93/RPSA/RPL3/RPL6/RPS4X/RPL23/RPS8/PCBP2/RPS18/RPL21/RPS15/NUP210/RPL5/EIF3F/RPL10/RPS5/RPS3A/DHX9/RPS19/RPL8/RPL27/RPL15/RPL12/NUP37/RPL14/PTBP1/RPS13/RPL24/RPL11/TARDBP/RPS28/RPS25/RPS26/EIF3G/RPL10A/RPS23/RPL36/RPS9/RPL28/RPL23A/SMARCA4/RAE1/RPL35/RPL30/RPS29
GO:0006413	translational initiation	1.09138E-31	50	EIF4B/EIF3B/EIF3A/RPSA/PABPC1/RPL3/RPL6/RPS4X/RPL23/RPS8/RPS18/RPL21/RPS15/EIF3I/EIF3M/DDX3X/RPL5/EIF3F/RPL10/RPS5/RPS3A/RPS19/EIF2S3/RPL8/PPP1CA/EIF3H/RPL27/RPL15/RPL12/RPL14/RPS13/RPL24/RPL11/RPS28/RPS25/RPS26/EIF3G/EIF2B3/RPL10A/RPS23/RPL36/RPS9/RPL28/EIF5/RPL23A/EIF2B5/RPL35/CDC123/RPL30/RPS29
GO:0002479	antigen processing and presentation of exogenous peptide antigen via MHC class I, TAP-dependent	1.01826E-28	33	PSMD2/PSMD1/PSMC3/PSMC4/PSMC5/PSMC1/PSMC2/PSMD13/PSMC6/PSMD12/PSMA2/PSMD7/PSMD11/PSMB4/PSMD14/HLA-A/PSMB1/PSMB3/PSMA5/PSMA7/PSMA1/PSMA4/CALR/PSMB7/PSMD8/PSMD4/PSMA6/PSMA3/PDIA3/PSMB2/PSME3/SEC22B/PSMB6

GO:0072599	establishment of protein localization to endoplasmic reticulum	5.27351E-28	39	HSPA5/RPSA/RPL3/RPL6/RPS4X/RPL23/RPS8/RPS18/RPL21/RPS15/SEC61A1/RPL5/RPL10/RPS5/RPS3A/RPS19/RPL8/SPCS2/RPL27/RPL15/RPL12/RPL14/RPS13/RAB10/RPL24/RPL11/RPS28/RPS25/RPS26/RPL10A/RPS23/RPL36/RPS9/RPL28/RPL23A/RPL35/SEC61B/RPL30/RPS29
GO:0019083	viral transcription	7.51199E-28	45	PSMC3/NUP188/NUP160/RANBP2/NUP93/RPSA/RPL3/RPL6/RPS4X/RPL23/RPS8/RPS18/RPL21/RPS15/NUP210/RPL5/RPL10/RPS5/RPS3A/DHX9/RPS19/RPL8/RPL27/RPL15/RPL12/NUP37/RPL14/RPS13/RPL24/RPL11/TARDBP/RPS28/RPS25/RPS26/RPL10A/RPS23/RPL36/RPS9/RPL28/RPL23A/SMARCA4/RAE1/RPL35/RPL30/RPS29
GO:0042590	antigen processing and presentation of exogenous peptide antigen via MHC class I	8.49369E-28	33	PSMD2/PSMD1/PSMC3/PSMC4/PSMC5/PSMC1/PSMC2/PSMD13/PSMC6/PSMD12/PSMA2/PSMD7/PSMD11/PSMB4/PSMD14/HLA-A/PSMB1/PSMB3/PSMA5/PSMA7/PSMA1/PSMA4/CALR/PSMB7/PSMD8/PSMD4/PSMA6/PSMA3/PDIA3/PSMB2/PSME3/SEC22B/PSMB6
GO:0006613	cotranslational protein targeting to membrane	1.57293E-27	37	RPSA/RPL3/RPL6/RPS4X/RPL23/RPS8/RPS18/RPL21/RPS15/SEC61A1/RPL5/RPL10/RPS5/RPS3A/RPS19/RPL8/RPL27/RPL15/SSR1/RPL12/RPL14/RPS13/RPL24/RPL11/RPS28/RPS25/RPS26/RPL10A/RPS23/RPL36/RPS9/RPL28/RPL23A/RPL35/SEC61B/RPL30/RPS29
GO:0045047	protein targeting to ER	2.33952E-27	38	HSPA5/RPSA/RPL3/RPL6/RPS4X/RPL23/RPS8/RPS18/RPL21/RPS15/SEC61A1/RPL5/RPL10/RPS5/RPS3A/RPS19/RPL8/SPCS2/RPL27/RPL15/RPL12/RPL14/RPS13/RPL24/RPL11/RPS28/RPS25/RPS26/RPL10A/RPS23/RPL36/RPS9/RPL28/RPL23A/RPL35/SEC61B/RPL30/RPS29
GO:0006614	SRP-dependent cotranslational protein targeting to membrane	6.76412E-27	36	RPSA/RPL3/RPL6/RPS4X/RPL23/RPS8/RPS18/RPL21/RPS15/SEC61A1/RPL5/RPL10/RPS5/RPS3A/RPS19/RPL8/RPL27/RPL15/RPL12/RPL14/RPS13/RPL24/RPL11/RPS28/RPS25/RPS26/RPL10A/RPS23/RPL36/RPS9/RPL28/RPL23A/RPL35/SEC61B/RPL30/RPS29
GO:0070498	interleukin-1-mediated signaling pathway	1.93461E-26	35	PSMD2/OTUD4/PSMD1/PSMC3/PSMC4/PSMC5/PSMC1/PSMC2/PSMD13/PSMC6/PSMD12/MAP3K7/PSMA2/PSMD7/PSMD11/PSMB4/PSMD14/PSMB1/PSMB3/TAB2/SQSTM1/TAB3/PSMA5/PSMA7/PSMA1/PSMA4/PSMB7/PSMD8/PSMD4/PSMA6/PSMA3/PSMB2/PSME3/PSMB6/SKP1
GO:0006521	regulation of cellular amino acid metabolic process	6.48498E-26	29	PSMD2/PSMD1/PSMC3/PSMC4/PSMC5/PSMC1/PSMC2/PSMD13/PSMC6/PSMD12/PSMA2/PSMD7/PSMD11/PSMB4/PSMD14/PSMB1/PSMB3/PSMA5/PSMA7/PSMA1/PSMA4/PSMB7/PSMD8/PSMD4/PSMA6/PSMA3/PSMB2/PSME3/PSMB6
GO:0002474	antigen processing and presentation of peptide antigen via MHC class I	8.1474E-26	34	PSMD2/CANX/PSMD1/PSMC3/PSMC4/PSMC5/PSMC1/PSMC2/PSMD13/PSMC6/PSMD12/PSMA2/PSMD7/PSMD11/PSMB4/PSMD14/HLA-A/PSMB1/PSMB3/PSMA5/PSMA7/PSMA1/PSMA4/CALR/PSMB7/PSMD8/PSMD4/PSMA6/PSMA3/PDIA3/PSMB2/PSME3/SEC22B/PSMB6
GO:0070972	protein localization to endoplasmic reticulum	1.01813E-25	40	HSPA5/RPSA/RPL3/UBAC2/RPL6/RPS4X/RPL23/RPS8/RPS18/RPL21/RPS15/SEC61A1/RPL5/RPL10/RPS5/RPS3A/RPS19/RPL8/SPCS2/RPL27/RPL15/RPL12/RPL14/RPS13/RAB10/RPL24/RPL11/RPS28/RPS25/RPS26/RPL10A/RPS23/RPL36/RPS9/RPL28/RPL23A/RPL35/SEC61B/RPL30/RPS29

GO:0022618	ribonucleoprotein complex assembly	1.07607E-24	51	PRMT5/EIF4B/PRPF31/WDR77/SNRNP200/HSP90AB1/HSP90AA1/DDX20/CLNS1A/EIF3B/PRPF8/GEMIN4/SF3B1/EIF3A/RPSA/RPL3/RPL6/STRAP/AAR2/RPS15/EIF3I/SMN1/SNRPD2/EIF3M/SNRPB/DDX3X/RPL5/EIF3F/RPL10/RPS5/DHX9/RPS19/EIF2S3/EIF3H/SNRPD3/RPL12/GEMIN5/RPL24/RPL11/RPS28/RPS27L/EIF3G/RPS23/GEMIN6/CNOT1/SNRPF/EIF5/RPL23A/SNRPG/SNRPE/PTGES3
GO:0071826	ribonucleoprotein complex subunit organization	1.39832E-24	52	PRMT5/VCP/EIF4B/PRPF31/WDR77/SNRNP200/HSP90AB1/HSP90AA1/DDX20/CLNS1A/EIF3B/PRPF8/GEMIN4/SF3B1/EIF3A/RPSA/RPL3/RPL6/STRAP/AAR2/RPS15/EIF3I/SMN1/SNRPD2/EIF3M/SNRPB/DDX3X/RPL5/EIF3F/RPL10/RPS5/DHX9/RPS19/EIF2S3/EIF3H/SNRPD3/RPL12/GEMIN5/RPL24/RPL11/RPS28/RPS27L/EIF3G/RPS23/GEMIN6/CNOT1/SNRPF/EIF5/RPL23A/SNRPG/SNRPE/PTGES3
GO:0000184	nuclear-transcribed mRNA catabolic process, nonsense-mediated decay	1.49484E-24	36	RPSA/PABPC1/RPL3/PPP2R1A/RPL6/RPS4X/RPL23/RPS8/RPS18/RPL21/RPS15/RPL5/RPL10/RPS5/RPS3A/RPS19/RPL8/RPL27/RPL15/RPL12/RPL14/RPS13/RPL24/RPL11/RPS28/RPS25/RPS26/RPL10A/RPS23/RPL36/RPS9/RPL28/RPL23A/RPL35/RPL30/RPS29
GO:0031145	anaphase-promoting complex-dependent catabolic process	1.51979E-24	31	PSMD2/PSMD1/PSMC3/PSMC4/PSMC5/PSMC1/PSMC2/PSMD13/PSMC6/PSMD12/PSMA2/PSMD7/PSMD11/PSMB4/PSMD14/PSMB1/PSMB3/PSMA5/PSMA7/PSMA1/PSMA4/PSMB7/PSMD8/PSMD4/PSMA6/PSMA3/PSMB2/CDK1/PSME3/PSMB6/PLK1
GO:0090150	establishment of protein localization to membrane	1.94617E-24	55	HSPA5/SPTBN1/BAG6/HSP90AA1/NMT1/STOM/RPSA/RPL3/RPL6/RACK1/RPS4X/GET4/RPL23/RPS8/RPS18/RPL21/RPS15/SEC61A1/RPL5/UBL4A/RPL10/SAMM50/RPS5/RPS3A/RPS19/RPL8/RPL27/CHP1/RPL15/SSR1/AGK/RPL12/MFF/RPL14/RPS13/RAB10/RPL24/RPL11/EXOC4/RPS28/RPS25/RPS26/TFDP1/RPL10A/RPS23/NDUFA13/RPL36/PEX3/RPS9/RPL28/RPL23A/RPL35/SEC61B/RPL30/RPS29
GO:0010498	proteasomal protein catabolic process	2.39351E-24	65	VCP/HSPA5/PSMD2/PSMD1/PSMC3/HSP90AB1/BAG6/PSMC4/PSMC5/PSMC1/PSMC2/FAF2/PSMD13/PSMC6/ECPAS/PSMD12/NPLOC4/ERLIN2/PSMA2/PSMD7/PSMD11/PSMB4/PSMD14/ERLIN1/RAD23B/PSMB1/LTN1/HSP90B1/DDB1/PSMB3/UBAC2/PSMA5/RACK1/PSMA7/PSMA1/STT3B/PSMA4/GET4/PSMB7/PSMD8/CCDC47/PSMD4/PCBP2/AMFR/PSMA6/PSMA3/AUP1/HM13/UFD1/FBXW5/PSMB2/CDK1/PSME3/PSMB6/SKP1/PRKACA/CLU/OGT/RPL11/STUB1/CAV1/DERL1/TMTC3/SEC61B/PLK1
GO:0061418	regulation of transcription from RNA polymerase II promoter in response to hypoxia	5.94988E-24	30	PSMD2/PSMD1/PSMC3/PSMC4/PSMC5/PSMC1/PSMC2/PSMD13/PSMC6/PSMD12/PSMA2/PSMD7/PSMD11/PSMB4/PSMD14/PSMB1/PSMB3/PSMA5/PSMA7/PSMA1/PSMA4/PSMB7/PSMD8/PSMD4/PSMA6/PSMA3/PSMB2/PSME3/ELOC/PSMB6

GO:0002478	antigen processing and presentation of exogenous peptide antigen	1.26875E-23	41	PSMD2/CANX/PSMD1/PSMC3/PSMC4/PSMC5/PSMC1/PSMC2/PSMD13/PSMC6/PSMD12/PSMA2/PSMD7/PSMD11/PSMB4/PSMD14/HLA-A/PSMB1/PSMB3/PSMA5/CLTC/PSMA7/PSMA1/PSMA4/CALR/PSMB7/PSMD8/PSMD4/ACTR1A/PSMA6/PSMA3/PDIA3/PSMB2/PSME3/SEC22B/PSMB6/DYNC1I2/KIF5A/DCTN2/CAPZA1/RAB7A
GO:1902036	regulation of hematopoietic stem cell differentiation	1.41408E-23	29	PSMD2/PSMD1/PSMC3/PSMC4/PSMC5/PSMC1/PSMC2/PSMD13/PSMC6/PSMD12/PSMA2/PSMD7/PSMD11/PSMB4/PSMD14/PSMB1/PSMB3/PSMA5/PSMA7/PSMA1/PSMA4/PSMB7/PSMD8/PSMD4/PSMA6/PSMA3/PSMB2/PSME3/PSMB6
GO:0043161	proteasome-mediated ubiquitin-dependent protein catabolic process	2.10569E-23	60	VCP/HSPA5/PSMD2/PSMD1/PSMC3/HSP90AB1/BAG6/PSMC4/PSMC5/PSMC1/PSMC2/FAF2/PSMD13/PSMC6/ECPAS/PSMD12/NPLOC4/ERLIN2/PSMA2/PSMD7/PSMD11/PSMB4/PSMD14/ERLIN1/RAD23B/PSMB1/LTN1/HSP90B1/DDB1/PSMB3/PSMA5/RACK1/PSMA7/PSMA1/STT3B/PSMA4/PSMB7/PSMD8/CCDC47/PSMD4/PCBP2/AMFR/PSMA6/PSMA3/AUP1/UFD1/FBXW5/PSMB2/CDK1/PSME3/PSMB6/SKP1/CLU/OGT/RPL11/STUB1/CAV1/DERL1/SEC61B/PLK1
GO:0061013	regulation of mRNA catabolic process	2.32655E-23	43	XPO1/PSMD2/PSMD1/PSMC3/PSMC4/PSMC5/PSMC1/PSMC2/PSMD13/PSMC6/PSMD12/TNRC6B/PSMA2/PSMD7/PSMD11/PSMB4/PSMD14/HNRNPU/PSMB1/PSMB3/PABPC1/PSMA5/PSMA7/PSMA1/HNRNPM/PSMA4/PSMB7/PSMD8/THRAP3/PABPC4/PSMD4/PSMA6/PSMA3/ZC3HAV1/PSMB2/DHX9/PSME3/PSMB6/HNRNPA0/TARDBP/VIM/CNOT1/HNRNPD
GO:0002223	stimulatory C-type lectin receptor signaling pathway	4.23803E-23	34	PSMD2/PSMD1/PSMC3/PSMC4/PSMC5/PSMC1/PSMC2/PSMD13/PSMC6/PSMD12/MAP3K7/PSMA2/PSMD7/PSMD11/PSMB4/PSMD14/PSMB1/PSMB3/TAB2/TAB3/PSMA5/PSMA7/PSMA1/PSMA4/PSMB7/PSMD8/PSMD4/PSMA6/PSMA3/PSMB2/PSME3/PSMB6/SKP1/PRKACA
GO:0006612	protein targeting to membrane	4.45142E-23	43	HSPA5/STOM/RPSA/RPL3/RPL6/RPS4X/RPL23/RPS8/RPS18/RPL21/RPS15/SEC61A1/RPL5/RPL10/RPS5/RPS3A/RPS19/RPL8/RPL27/CHP1/RPL15/SSR1/RPL12/MFF/RPL14/RPS13/RPL24/RPL11/EXOC4/RPS28/RPS25/RPS26/RPL10A/RPS23/RPL36/PEX3/RPS9/RPL28/RPL23A/RPL35/SEC61B/RPL30/RPS29
GO:0019884	antigen processing and presentation of exogenous antigen	6.60263E-23	41	PSMD2/CANX/PSMD1/PSMC3/PSMC4/PSMC5/PSMC1/PSMC2/PSMD13/PSMC6/PSMD12/PSMA2/PSMD7/PSMD11/PSMB4/PSMD14/HLA-A/PSMB1/PSMB3/PSMA5/CLTC/PSMA7/PSMA1/PSMA4/CALR/PSMB7/PSMD8/PSMD4/ACTR1A/PSMA6/PSMA3/PDIA3/PSMB2/PSME3/SEC22B/PSMB6/DYNC1I2/KIF5A/DCTN2/CAPZA1/RAB7A

GO:0006605	protein targeting	1.11117E-22	60	HUWE1/HSPA5/GCC2/HSP90AA1/LMAN1/STOM/RPSA/RPL3/TOMM40/RPL6/VPS13A/USP9X/RPS4X/HAX1/RPL23/TIMM50/RPS8/RPS18/RPL21/RPS15/TIMM23B/SEC61A1/RPL5/RPL10/SAMM50/RPS5/RPS3A/RPS19/RPL8/SPCS2/RPL27/CHP1/RPL15/SSR1/AGK/RPL12/MFF/RPL14/CLU/RPS13/RPL24/RPL11/EXOC4/RPS28/RPS25/RPS26/AKAP12/RPL10A/RPS23/NDUFA13/RPL36/PEX3/RPS9/RPL28/RPL23A/RAB7A/RPL35/SEC61B/RPL30/RPS29
GO:0002220	innate immune response activating cell surface receptor signaling pathway	1.1258E-22	34	PSMD2/PSMD1/PSMC3/PSMC4/PSMC5/PSMC1/PSMC2/PSMD13/PSMC6/PSMD12/MAP3K7/PSMA2/PSMD7/PSMD11/PSMB4/PSMD14/PSMB1/PSMB3/TAB2/TAB3/PSMA5/PSMA7/PSMA1/PSMA4/PSMB7/PSMD8/PSMD4/PSMA6/PSMA3/PSMB2/PSME3/PSMB6/SKP1/PRKACA
GO:0010972	negative regulation of G2/M transition of mitotic cell cycle	2.17526E-22	31	PSMD2/PSMD1/PSMC3/PSMC4/PSMC5/PSMC1/PSMC2/PSMD13/PSMC6/PSMD12/PSMA2/PSMD7/PSMD11/PSMB4/PSMD14/PSMB1/PSMB3/PSMA5/PSMA7/PSMA1/PSMA4/PSMB7/PSMD8/PSMD4/PSMA6/PSMA3/PSMB2/CDK1/PSME3/PSMB6/SKP1
GO:0043488	regulation of mRNA stability	2.42291E-22	40	XPO1/PSMD2/PSMD1/PSMC3/PSMC4/PSMC5/PSMC1/PSMC2/PSMD13/PSMC6/PSMD12/PSMA2/PSMD7/PSMD11/PSMB4/PSMD14/HNRNPU/PSMB1/PSMB3/PABPC1/PSMA5/PSMA7/PSMA1/HNRNPM/PSMA4/PSMB7/PSMD8/THRAP3/PABPC4/PSMD4/PSMA6/PSMA3/PSMB2/DHX9/PSME3/PSMB6/HNRNPA0/TARDBP/VIM/HNRNPD
GO:0048002	antigen processing and presentation of peptide antigen	3.17933E-22	41	PSMD2/CANX/PSMD1/PSMC3/PSMC4/PSMC5/PSMC1/PSMC2/PSMD13/PSMC6/PSMD12/PSMA2/PSMD7/PSMD11/PSMB4/PSMD14/HLA-A/PSMB1/PSMB3/PSMA5/CLTC/PSMA7/PSMA1/PSMA4/CALR/PSMB7/PSMD8/PSMD4/ACTR1A/PSMA6/PSMA3/PDIA3/PSMB2/PSME3/SEC22B/PSMB6/DYNC1I2/KIF5A/DCTN2/CAPZA1/RAB7A
GO:0031146	SCF-dependent proteasomal ubiquitin-dependent protein catabolic process	4.58311E-22	31	PSMD2/PSMD1/PSMC3/PSMC4/PSMC5/PSMC1/PSMC2/PSMD13/PSMC6/PSMD12/PSMA2/PSMD7/PSMD11/PSMB4/PSMD14/PSMB1/PSMB3/PSMA5/PSMA7/PSMA1/PSMA4/PSMB7/PSMD8/PSMD4/PSMA6/PSMA3/FBXW5/PSMB2/PSME3/PSMB6/SKP1
GO:0033238	regulation of cellular amine metabolic process	8.01037E-22	29	PSMD2/PSMD1/PSMC3/PSMC4/PSMC5/PSMC1/PSMC2/PSMD13/PSMC6/PSMD12/PSMA2/PSMD7/PSMD11/PSMB4/PSMD14/PSMB1/PSMB3/PSMA5/PSMA7/PSMA1/PSMA4/PSMB7/PSMD8/PSMD4/PSMA6/PSMA3/PSMB2/PSME3/PSMB6
GO:1902750	negative regulation of cell cycle G2/M phase transition	8.54181E-22	32	PSMD2/PSMD1/PSMC3/PSMC4/PSMC5/PSMC1/PSMC2/PSMD13/PSMC6/PSMD12/PSMA2/PSMD7/PSMD11/PSMB4/PSMD14/PSMB1/PSMB3/PSMA5/PSMA7/PSMA1/PSMA4/PSMB7/PSMD8/PSMD4/PSMA6/PSMA3/PSMB2/CDK1/PSME3/PSMB6/SKP1/PLK1

GO:0043487	regulation of RNA stability	9.40132E-22	40	XPO1/PSMD2/PSMD1/PSMC3/PSMC4/PSMC5/PSMC1/PSMC2/PSMD13/PSMC6/PSMD12/PSMA2/PSMD7/PSMD11/PSMB4/PSMD14/HNRNPU/PSMB1/PSMB3/PABPC1/PSMA5/PSMA7/PSMA1/HNRNPM/PSMA4/PSMB7/PSMD8/THRAP3/PABPC4/PSMD4/PSMA6/PSMA3/PSMB2/DHX9/PSME3/PSMB6/HNRNPA0/TARDBP/VIM/HNRNPD
GO:0010389	regulation of G2/M transition of mitotic cell cycle	1.42529E-21	41	PSMD2/PSMD1/PSMC3/PSMC4/PSMC5/PSMC1/PSMC2/HSP90AA1/PSMD13/PSMC6/PSMD12/PSMA2/PSMD7/PSMD11/PSMB4/PSMD14/PSMB1/CEP250/PSMB3/CKAP5/PPP2R1A/PSMA5/PSMA7/PSMA1/PSMA4/PSMB7/PSMD8/PSMD4/ACTR1A/PSMA6/PSMA3/PSMB2/CDK1/PSME3/PSMB6/DYNC1I2/SKP1/PRKACA/NDE1/DCTN2/PLK1
GO:0060218	hematopoietic stem cell differentiation	1.81151E-21	29	PSMD2/PSMD1/PSMC3/PSMC4/PSMC5/PSMC1/PSMC2/PSMD13/PSMC6/PSMD12/PSMA2/PSMD7/PSMD11/PSMB4/PSMD14/PSMB1/PSMB3/PSMA5/PSMA7/PSMA1/PSMA4/PSMB7/PSMD8/PSMD4/PSMA6/PSMA3/PSMB2/PSME3/PSMB6
GO:1903311	regulation of mRNA metabolic process	2.17375E-21	51	XPO1/PSMD2/PSMD1/PSMC3/PSMC4/PSMC5/PSMC1/HNRNPK/PSMC2/PSMD13/PSMC6/PSMD12/TNRC6B/PSMA2/PSMD7/PSMD11/PSMB4/PSMD14/HNRNPU/PSMB1/PSMB3/PABPC1/PSMA5/PSMA7/PSMA1/HNRNPM/PSMA4/PSMB7/PSMD8/THRAP3/PABPC4/PSMD4/HNRNPL/PSMA6/PSMA3/PRDX6/ZC3HAV1/HNRNPA1/PSMB2/DHX9/PSME3/PSMB6/SNRNP70/HNRNPA0/PTBP1/TARDBP/VIM/CNOT1/HNRNPD/NSRP1/PABPN1
GO:1901532	regulation of hematopoietic progenitor cell differentiation	3.99565E-21	29	PSMD2/PSMD1/PSMC3/PSMC4/PSMC5/PSMC1/PSMC2/PSMD13/PSMC6/PSMD12/PSMA2/PSMD7/PSMD11/PSMB4/PSMD14/PSMB1/PSMB3/PSMA5/PSMA7/PSMA1/PSMA4/PSMB7/PSMD8/PSMD4/PSMA6/PSMA3/PSMB2/PSME3/PSMB6
GO:0019882	antigen processing and presentation	5.35522E-21	43	PSMD2/CANX/PSMD1/PSMC3/PSMC4/PSMC5/PSMC1/PSMC2/PSMD13/PSMC6/PSMD12/PSMA2/PSMD7/PSMD11/PSMB4/PSMD14/HLA-A/PSMB1/PSMB3/PSMA5/CLTC/PSMA7/PSMA1/PSMA4/CALR/PSMB7/PSMD8/PSMD4/ACTR1A/PSMA6/PSMA3/PDIA3/PSMB2/PSME3/SEC22B/PSMB6/DYNC1I2/RAB10/KIF5A/DCTN2/CAPZA1/RAB7A/RAB32
GO:0043618	regulation of transcription from RNA polymerase II promoter in response to stress	8.06561E-21	33	GCN1/HSPA5/PSMD2/PSMD1/PSMC3/PSMC4/PSMC5/PSMC1/PSMC2/PSMD13/PSMC6/PSMD12/PSMA2/PSMD7/PSMD11/PSMB4/PSMD14/PSMB1/PSMB3/PSMA5/PSMA7/PSMA1/PSMA4/PSMB7/PSMD8/PSMD4/DNAJB1/PSMA6/PSMA3/PSMB2/PSME3/ELOC/PSMB6
GO:1902749	regulation of cell cycle G2/M phase transition	4.18987E-20	41	PSMD2/PSMD1/PSMC3/PSMC4/PSMC5/PSMC1/PSMC2/HSP90AA1/PSMD13/PSMC6/PSMD12/PSMA2/PSMD7/PSMD11/PSMB4/PSMD14/PSMB1/CEP250/PSMB3/CKAP5/PPP2R1A/PSMA5/PSMA7/PSMA1/PSMA4/PSMB7/PSMD8/PSMD4/ACTR1A/PSMA6/PSMA3/PSMB2/CDK1/PSME3/PSMB6/DYNC1I2/SKP1/PRKACA/NDE1/DCTN2/PLK1

GO:0043620	regulation of DNA-templated transcription in response to stress	4.41764E-20	33	GCN1/HSPA5/PSMD2/PSMD1/PSMC3/PSMC4/PSMC5/PSMC1/PSMC2/PSMD13/PSMC6/PSMD12/PSMA2/PSMD7/PSMD11/PSMB4/PSMD14/PSMB1/PSMB3/PSMA5/PSMA7/PSMA1/PSMA4/PSMB7/PSMD8/PSMD4/DNAJB1/PSMA6/PSMA3/PSMB2/PSME3/ELOC/PSMB6
GO:2000736	regulation of stem cell differentiation	4.03475E-19	31	PSMD2/PSMD1/PSMC3/PSMC4/PSMC5/PSMC1/PSMC2/PSMD13/PSMC6/PSMD12/PSMA2/PSMD7/PSMD11/PSMB4/PSMD14/HNRNPU/PSMB1/PSMB3/PSMA5/PSMA7/PSMA1/PSMA4/PSMB7/PSMD8/PSMD4/PSMA6/PSMA3/PSMB2/PSME3/PSMB6/SEMA3C
GO:0030177	positive regulation of Wnt signaling pathway	4.94668E-19	37	VCP/PSMD2/PSMD1/PSMC3/PSMC4/PSMC5/PSMC1/PSMC2/PSMD13/PSMC6/PSMD12/PSMA2/PSMD7/PSMD11/PSMB4/PSMD14/PSMB1/SPIN1/PSMB3/PSMA5/PSMA7/PSMA1/UBR5/PSMA4/ATP6AP2/PSMB7/PSMD8/PSMD4/PSMA6/PSMA3/DDX3X/PSMB2/PSME3/PSMB6/CAV1/SMARCA4/WLS
GO:0090263	positive regulation of canonical Wnt signaling pathway	5.68463E-19	34	VCP/PSMD2/PSMD1/PSMC3/PSMC4/PSMC5/PSMC1/PSMC2/PSMD13/PSMC6/PSMD12/PSMA2/PSMD7/PSMD11/PSMB4/PSMD14/PSMB1/PSMB3/PSMA5/PSMA7/PSMA1/UBR5/PSMA4/PSMB7/PSMD8/PSMD4/PSMA6/PSMA3/DDX3X/PSMB2/PSME3/PSMB6/CAV1/WLS
GO:0060071	Wnt signaling pathway, planar cell polarity pathway	5.74275E-19	30	PSMD2/PSMD1/PSMC3/PSMC4/PSMC5/PSMC1/PSMC2/PSMD13/PSMC6/PSMD12/PSMA2/PSMD7/PSMD11/PSMB4/PSMD14/PSMB1/PSMB3/PSMA5/CLTC/PSMA7/PSMA1/PSMA4/PSMB7/PSMD8/PSMD4/PSMA6/PSMA3/PSMB2/PSME3/PSMB6
GO:0090175	regulation of establishment of planar polarity	1.03692E-18	30	PSMD2/PSMD1/PSMC3/PSMC4/PSMC5/PSMC1/PSMC2/PSMD13/PSMC6/PSMD12/PSMA2/PSMD7/PSMD11/PSMB4/PSMD14/PSMB1/PSMB3/PSMA5/CLTC/PSMA7/PSMA1/PSMA4/PSMB7/PSMD8/PSMD4/PSMA6/PSMA3/PSMB2/PSME3/PSMB6
GO:0000956	nuclear-transcribed mRNA catabolic process	1.20335E-18	39	TNRC6B/RPSA/PABPC1/RPL3/PPP2R1A/RPL6/RPS4X/RPL23/THRAP3/RPS8/RPS18/RPL21/RPS15/RPL5/RPL10/RPS5/RPS3A/RPS19/RPL8/RPL27/RPL15/RPL12/RPL14/RPS13/RPL24/RPL11/RPS28/RPS25/RPS26/RPL10A/RPS23/RPL36/CNOT1/RPS9/RPL28/RPL23A/RPL35/RPL30/RPS29
GO:0062012	regulation of small molecule metabolic process	3.52555E-18	56	VCP/PSMD2/KPNB1/PSMD1/PSMC3/PSMC4/FASN/PSMC5/PSMC1/PSMC2/NUP188/PSMD13/NUP160/PSMC6/PSMD12/ERLIN2/RANBP2/PSMA2/PSMD7/PSMD11/PSMB4/PSMD14/ERLIN1/PSMB1/NUP93/PFKFB3/CYP51A1/DDB1/PSMB3/PSMA5/CLTC/PSMA7/PSMA1/PSMA4/PSMB7/PSMD8/DHCR7/PSMD4/NUP210/PSMA6/PSMA3/FDFT1/PSMB2/CDK1/PSME3/PPP1CA/ZMPSTE24/PSMB6/RAN/NUP37/OGT/SCD/CAV1/SQLE/RAE1/FAM3A
GO:0071347	cellular response to interleukin-1	5.01711E-18	36	PSMD2/OTUD4/PSMD1/PSMC3/PSMC4/PSMC5/PSMC1/PSMC2/PSMD13/PSMC6/PSMD12/MAP3K7/PSMA2/PSMD7/PSMD11/PSMB4/PSMD14/PSMB1/PSMB3/TAB2/SQSTM1/TAB3/PSMA5/PSMA7/PSMA1/PSMA4/PSMB7/PSMD8/PSMD4/PSMA6/PSMA3/PSMB2/PSME3/PSMB6/SKP1/AKAP12

GO:0000086	G2/M transition of mitotic cell cycle	1.44519E-17	41	PSMD2/PSMD1/PSMC3/PSMC4/PSMC5/PSMC1/PSMC2/HSP90AA1/PSMD13/PSMC6/PSMD12/PSMA2/PSMD7/PSMD11/PSMB4/PSMD14/PSMB1/CEP250/PSMB3/CKAP5/PPP2R1A/PSMA5/PSMA7/PSMA1/PSMA4/PSMB7/PSMD8/PSMD4/ACTR1A/PSMA6/PSMA3/PSMB2/CDK1/PSME3/PSMB6/DYNC1I2/SKP1/PRKACA/NDE1/DCTN2/PLK1
GO:0001738	morphogenesis of a polarized epithelium	1.90806E-17	32	PSMD2/PSMD1/PSMC3/PSMC4/PSMC5/PSMC1/PSMC2/PSMD13/PSMC6/PSMD12/PSMA2/PSMD7/PSMD11/PSMB4/PSMD14/PSMB1/PSMB3/PSMA5/CLTC/PSMA7/PSMA1/PSMA4/PSMB7/PSMD8/PSMD4/PSMA6/PSMA3/PSMB2/CAMSAP3/PSME3/PSMB6/RAB10
GO:0044839	cell cycle G2/M phase transition	3.3517E-17	42	PSMD2/PSMD1/PSMC3/PSMC4/PSMC5/PSMC1/PSMC2/HSP90AA1/PSMD13/PSMC6/PSMD12/PSMA2/PSMD7/PSMD11/PSMB4/PSMD14/PSMB1/CEP250/PSMB3/CKAP5/PPP2R1A/PSMA5/PSMA7/PSMA1/AKAP8L/PSMA4/PSMB7/PSMD8/PSMD4/ACTR1A/PSMA6/PSMA3/PSMB2/CDK1/PSME3/PSMB6/DYNC1I2/SKP1/PRKACA/NDE1/DCTN2/PLK1
GO:0001736	establishment of planar polarity	4.57798E-17	30	PSMD2/PSMD1/PSMC3/PSMC4/PSMC5/PSMC1/PSMC2/PSMD13/PSMC6/PSMD12/PSMA2/PSMD7/PSMD11/PSMB4/PSMD14/PSMB1/PSMB3/PSMA5/CLTC/PSMA7/PSMA1/PSMA4/PSMB7/PSMD8/PSMD4/PSMA6/PSMA3/PSMB2/PSME3/PSMB6
GO:0007164	establishment of tissue polarity	4.57798E-17	30	PSMD2/PSMD1/PSMC3/PSMC4/PSMC5/PSMC1/PSMC2/PSMD13/PSMC6/PSMD12/PSMA2/PSMD7/PSMD11/PSMB4/PSMD14/PSMB1/PSMB3/PSMA5/CLTC/PSMA7/PSMA1/PSMA4/PSMB7/PSMD8/PSMD4/PSMA6/PSMA3/PSMB2/PSME3/PSMB6
GO:0035567	non-canonical Wnt signaling pathway	1.4175E-16	32	PSMD2/PSMD1/PSMC3/PSMC4/PSMC5/PSMC1/PSMC2/PSMD13/PSMC6/PSMD12/MAP3K7/TNRC6B/PSMA2/PSMD7/PSMD11/PSMB4/PSMD14/PSMB1/PSMB3/PSMA5/CLTC/PSMA7/PSMA1/PSMA4/PSMB7/PSMD8/PSMD4/PSMA6/PSMA3/PSMB2/PSME3/PSMB6
GO:0010948	negative regulation of cell cycle process	5.55966E-16	47	PRKDC/PSMD2/PSMD1/PSMC3/HSP90AB1/PSMC4/PSMC5/PSMC1/PSMC2/PSMD13/PSMC6/PSMD12/MSH6/PSMA2/PSMD7/PSMD11/PSMB4/PSMD14/MGA/PSMB1/PSMB3/ZW10/PSMA5/PSMA7/PSMA1/PSMA4/CALR/RPL23/PSMB7/PSMD8/PSMD4/PSMA6/PSMA3/PSMB2/PRKAR1A/CDK1/PSME3/TRIP13/PSMB6/SKP1/PRMT1/RPS27L/L3MBTL2/TFDP1/CNOT1/PCGF6/PLK1
GO:0038095	Fc-epsilon receptor signaling pathway	6.60743E-16	33	PSMD2/PSMD1/PSMC3/PSMC4/PSMC5/PSMC1/PSMC2/PSMD13/PSMC6/PSMD12/MAP3K7/PSMA2/PSMD7/PSMD11/PSMB4/PSMD14/PSMB1/PSMB3/TAB2/TAB3/PSMA5/PSMA7/PSMA1/PSMA4/PSMB7/PSMD8/PSMD4/PSMA6/PSMA3/PSMB2/PSME3/PSMB6/SKP1
GO:0070555	response to interleukin-1	8.13247E-16	36	PSMD2/OTUD4/PSMD1/PSMC3/PSMC4/PSMC5/PSMC1/PSMC2/PSMD13/PSMC6/PSMD12/MAP3K7/PSMA2/PSMD7/PSMD11/PSMB4/PSMD14/PSMB1/PSMB3/TAB2/SQSTM1/TAB3/PSMA5/PSMA7/PSMA1/PSMA4/PSMB7/PSMD8/PSMD4/PSMA6/PSMA3/PSMB2/PSME3/PSMB6/SKP1/AKAP12

GO:0038061	NIK/NF-kappaB signaling	9.61652E-16	34	PSMD2/PSMD1/PSMC3/PSMC4/PSMC5/PSMC1/PSMC2/PSMD13/PSMC6/PSMD12/MAP3K7/PSMA2/PSMD7/PSMD11/PSMB4/PSMD14/PSMB1/PSMB3/PSMA5/PPM1B/PSMA7/PSMA1/PSMA4/CALR/PSMB7/PSMD8/PSMD4/PSMA6/PSMA3/PRDX1/PSMB2/PSME3/PSMB6/SKP1
GO:1901991	negative regulation of mitotic cell cycle phase transition	9.89406E-16	39	PRKDC/PSMD2/PSMD1/PSMC3/PSMC4/PSMC5/PSMC1/PSMC2/PSMD13/PSMC6/PSMD12/PSMA2/PSMD7/PSMD11/PSMB4/PSMD14/PSMB1/PSMB3/ZW10/PSMA5/PSMA7/PSMA1/PSMA4/PSMB7/PSMD8/PSMD4/PSMA6/PSMA3/PSMB2/CDK1/PSME3/TRIP13/PSMB6/SKP1/PRMT1/RPS27L/TFDP1/CNOT1/PLK1
GO:0044106	cellular amine metabolic process	1.80834E-15	29	PSMD2/PSMD1/PSMC3/PSMC4/PSMC5/PSMC1/PSMC2/PSMD13/PSMC6/PSMD12/PSMA2/PSMD7/PSMD11/PSMB4/PSMD14/PSMB1/PSMB3/PSMA5/PSMA7/PSMA1/PSMA4/PSMB7/PSMD8/PSMD4/PSMA6/PSMA3/PSMB2/PSME3/PSMB6
GO:0050852	T cell receptor signaling pathway	2.95119E-15	35	PSMD2/PSMD1/PSMC3/PSMC4/PSMC5/PSMC1/PSMC2/PSMD13/PSMC6/PSMD12/MAP3K7/PSMA2/PSMD7/PSMD11/PSMB4/PSMD14/STOML2/PSMB1/PSMB3/TAB2/PSMA5/PSMA7/PSMA1/PSMA4/PSMB7/PSMD8/PSMD4/PSMA6/PSMA3/PSMB2/PSME3/PSMB6/SKP1/EIF2B3/EIF2B5
GO:0002244	hematopoietic progenitor cell differentiation	3.53605E-15	32	PRKDC/PSMD2/PSMD1/PSMC3/PSMC4/PSMC5/PSMC1/PSMC2/PSMD13/PSMC6/PSMD12/EEF2/PSMA2/PSMD7/PSMD11/PSMB4/PSMD14/PSMB1/PSMB3/PSMA5/PSMA7/PSMA1/PSMA4/ZBTB24/PSMB7/PSMD8/PSMD4/PSMA6/PSMA3/PSMB2/PSME3/PSMB6
GO:0071453	cellular response to oxygen levels	7.00472E-15	37	PSMD2/PSMD1/PSMC3/PSMC4/PSMC5/PSMC1/PSMC2/PSMD13/PSMC6/PSMD12/PSMA2/PSMD7/PSMD11/PSMB4/PSMD14/PSMB1/OPA1/PSMB3/PSMA5/PSMA7/PSMA1/ATP6AP1/PSMA4/PSMB7/PSMD8/NAMPT/PSMD4/AIFM1/PSMA6/PSMA3/PSMB2/PSME3/ELOC/PSMB6/STUB1/ATP6V1A/CAV1
GO:0008380	RNA splicing	7.74678E-15	52	PRMT5/PRPF31/WDR77/SNRNP200/HNRNPK/DDX20/CLNS1A/PRPF8/GEMIN4/SF3B1/EFTUD2/HNRNPF/HNRNPU/IVNS1ABP/SF3B3/PABPC1/PPP2R1A/AKAP8L/STRAP/HNRNPM/NONO/THRAP3/PCBP1/PCBP2/HNRNPL/AAR2/PRDX6/SMN1/SNRPD2/SF3B2/SNRPB/HNRNPA1/DHX9/SNRNP70/HNRNPA0/SNRPD3/SNRPA1/GEMIN5/PTBP1/RPS13/TARDBP/RPS26/GEMIN6/SF3B6/DHX15/HNRNPD/NSRP1/SNRPF/THOC3/SNRPG/SNRPE/PABPN1
GO:0009308	amine metabolic process	1.30058E-14	29	PSMD2/PSMD1/PSMC3/PSMC4/PSMC5/PSMC1/PSMC2/PSMD13/PSMC6/PSMD12/PSMA2/PSMD7/PSMD11/PSMB4/PSMD14/PSMB1/PSMB3/PSMA5/PSMA7/PSMA1/PSMA4/PSMB7/PSMD8/PSMD4/PSMA6/PSMA3/PSMB2/PSME3/PSMB6
GO:1901988	negative regulation of cell cycle phase transition	1.39922E-14	39	PRKDC/PSMD2/PSMD1/PSMC3/PSMC4/PSMC5/PSMC1/PSMC2/PSMD13/PSMC6/PSMD12/PSMA2/PSMD7/PSMD11/PSMB4/PSMD14/PSMB1/PSMB3/ZW10/PSMA5/PSMA7/PSMA1/PSMA4/PSMB7/PSMD8/PSMD4/PSMA6/PSMA3/PSMB2/CDK1/PSME3/TRIP13/PSMB6/SKP1/PRMT1/RPS27L/TFDP1/CNOT1/PLK1

GO:0006913	nucleocytoplasmic transport	1.50576E-14	44	IPO8/XPO1/IPO5/KPNB1/FLNA/XPOT/CSE1L/NUP188/NUP160/DDX20/TRIM28/RANBP2/GEMIN4/NUP93/EMD/RANBP6/AKAP8L/NF1/UBR5/CALR/RPL23/RANGAP1/RPS15/NUP210/SMN1/SNRPD2/SNRPB/HNRNPA1/DHX9/CHP1/RAN/SNRPD3/GEMIN5/PRKACA/NUP37/TARDBP/GEMIN6/SNRPF/RAE1/THOC3/TXN/SNRPG/SNRPE/PABPN1
GO:0016579	protein deubiquitination	1.68612E-14	40	VCP/PSMD2/OTUD4/PSMD1/PSMC3/PSMC4/PSMC5/PSMC1/PSMC2/PSMD13/PSMC6/PSMD12/MAP3K7/USP15/PSMA2/PSMD7/PSMD11/PSMB4/PSMD14/RAD23B/PSMB1/PSMB3/PSMA5/PSMA7/PSMA1/USP9X/PSMA4/PSMB7/PSMD8/PSMD4/PSMA6/PSMA3/UFD1/EIF3F/PSMB2/CDK1/ADRM1/PSME3/PSMB6/OGT
GO:1901990	regulation of mitotic cell cycle phase transition	1.89465E-14	50	PRKDC/PSMD2/PSMD1/PSMC3/PSMC4/PSMC5/PSMC1/PSMC2/HSP90AA1/PSMD13/PSMC6/PSMD12/PSMA2/PSMD7/PSMD11/PSMB4/PSMD14/PSMB1/CEP250/DDB1/PSMB3/ZW10/CKAP5/PPP2R1A/PSMA5/PSMA7/PSMA1/PSMA4/PSMB7/PSMD8/PSMD4/ACTR1A/PSMA6/PSMA3/DDX3X/PSMB2/CDK1/PSME3/TRIP13/PSMB6/DYNC1I2/SKP1/PRKACA/NDE1/PRMT1/DCTN2/RPS27L/TFDP1/CNOT1/PLK1
GO:0051169	nuclear transport	2.10589E-14	44	IPO8/XPO1/IPO5/KPNB1/FLNA/XPOT/CSE1L/NUP188/NUP160/DDX20/TRIM28/RANBP2/GEMIN4/NUP93/EMD/RANBP6/AKAP8L/NF1/UBR5/CALR/RPL23/RANGAP1/RPS15/NUP210/SMN1/SNRPD2/SNRPB/HNRNPA1/DHX9/CHP1/RAN/SNRPD3/GEMIN5/PRKACA/NUP37/TARDBP/GEMIN6/SNRPF/RAE1/THOC3/TXN/SNRPG/SNRPE/PABPN1
GO:0002181	cytoplasmic translation	3.29272E-14	25	EIF4B/EIF3B/EEF2/EIF3A/RPSA/RPL6/EIF3I/EIF3M/EIF3F/DHX9/EIF2S3/RPL8/EIF3H/RPL15/RPL24/RPL11/RPS28/RPS26/EIF3G/RPL10A/RPS23/RPL36/EIF5/RPL30/RPS29
GO:0002218	activation of innate immune response	3.42347E-14	42	PRKDC/PSMD2/OTUD4/PSMD1/PSMC3/PSMC4/PSMC5/PSMC1/PSMC2/PSMD13/PSMC6/PSMD12/NPLOC4/MAP3K7/PSMA2/PSMD7/PSMD11/PSMB4/PSMD14/PSMB1/HSP90B1/PSMB3/TAB2/TAB3/PSMA5/PSMA7/PSMA1/PSMA4/NONO/PSMB7/PSMD8/PSMD4/PSMA6/PSMA3/UFD1/PSMB2/PSME3/MATR3/PSMB6/SKP1/PRKACA/CAV1
GO:0010565	regulation of cellular ketone metabolic process	5.1522E-14	32	PSMD2/PSMD1/PSMC3/PSMC4/PSMC5/PSMC1/PSMC2/PSMD13/PSMC6/PSMD12/ERLIN2/PSMA2/PSMD7/PSMD11/PSMB4/PSMD14/ERLIN1/PSMB1/PSMB3/PSMA5/PSMA7/PSMA1/PSMA4/PSMB7/PSMD8/PSMD4/PSMA6/PSMA3/PSMB2/PSME3/PSMB6/CAV1
GO:0070646	protein modification by small protein removal	1.20852E-13	40	VCP/PSMD2/OTUD4/PSMD1/PSMC3/PSMC4/PSMC5/PSMC1/PSMC2/PSMD13/PSMC6/PSMD12/MAP3K7/USP15/PSMA2/PSMD7/PSMD11/PSMB4/PSMD14/RAD23B/PSMB1/PSMB3/PSMA5/PSMA7/PSMA1/USP9X/PSMA4/PSMB7/PSMD8/PSMD4/PSMA6/PSMA3/UFD1/EIF3F/PSMB2/CDK1/ADRM1/PSME3/PSMB6/OGT

GO:0038093	Fc receptor signaling pathway	1.35782E-13	36	PSMD2/PSMD1/PSMC3/HSP90AB1/PSMC4/PSMC5/PSMC1/PSMC2/HSP90AA1/PSMD13/PSMC6/PSMD12/MAP3K7/PSMA2/PSMD7/PSMD11/PSMB4/PSMD14/PSMB1/PSMB3/TAB2/TAB3/PSMA5/PSMA7/PSMA1/PSMA4/PSMB7/PSMD8/PSMD4/PSMA6/PSMA3/ELMO2/PSMB2/PSME3/PSMB6/SKP1
GO:0034976	response to endoplasmic reticulum stress	1.38849E-13	39	VCP/HSPA5/ATP2A2/CANX/BAG6/FAF2/PSMC6/ECPAS/NPLOC4/ERLIN2/EEF2/ERLIN1/OPA1/HSP90B1/UBAC2/RACK1/STT3B/GET4/CALR/CCDC47/AIFM1/AMFR/CLGN/AUP1/HM13/UFD1/PDIA3/SRPRB/TMEM33/SSR1/CLU/TARDBP/STUB1/TMCO1/CAV1/DERL1/TMTC3/EIF2B5/SEC61B
GO:0045089	positive regulation of innate immune response	1.54449E-13	45	PRKDC/PSMD2/OTUD4/PSMD1/PSMC3/PSMC4/PSMC5/PSMC1/PSMC2/PSMD13/PSMC6/PSMD12/NPLOC4/MAP3K7/PSMA2/PSMD7/PSMD11/PSMB4/PSMD14/PSMB1/HSP90B1/PSMB3/TAB2/TAB3/PSMA5/PSMA7/PSMA1/PSMA4/NONO/PSMB7/PSMD8/PLSCR1/PSMD4/PSMA6/PSMA3/UFD1/PSMB2/DHX9/RPS19/PSME3/MATR3/PSMB6/SKP1/PRKACA/CAV1
GO:0036294	cellular response to decreased oxygen levels	2.34391E-13	34	PSMD2/PSMD1/PSMC3/PSMC4/PSMC5/PSMC1/PSMC2/PSMD13/PSMC6/PSMD12/PSMA2/PSMD7/PSMD11/PSMB4/PSMD14/PSMB1/OPA1/PSMB3/PSMA5/PSMA7/PSMA1/PSMA4/PSMB7/PSMD8/NAMPT/PSMD4/AIFM1/PSMA6/PSMA3/PSMB2/PSME3/ELOC/PSMB6/STUB1
GO:0090090	negative regulation of canonical Wnt signaling pathway	2.60552E-13	31	PSMD2/PSMD1/PSMC3/PSMC4/PSMC5/PSMC1/PSMC2/PSMD13/PSMC6/PSMD12/PSMA2/PSMD7/PSMD11/PSMB4/PSMD14/PSMB1/EMD/PSMB3/PSMA5/PSMA7/PSMA1/PSMA4/PSMB7/PSMD8/PSMD4/PSMA6/PSMA3/PSMB2/PSME3/PSMB6/CAV1
GO:0033209	tumor necrosis factor-mediated signaling pathway	3.41444E-13	30	PSMD2/PSMD1/PSMC3/PSMC4/PSMC5/PSMC1/PSMC2/PSMD13/PSMC6/PSMD12/PSMA2/PSMD7/PSMD11/PSMB4/PSMD14/PSMB1/PSMB3/PSMA5/RACK1/PSMA7/PSMA1/PSMA4/PSMB7/PSMD8/PSMD4/PSMA6/PSMA3/PSMB2/PSME3/PSMB6
GO:1901987	regulation of cell cycle phase transition	4.86463E-13	50	PRKDC/PSMD2/PSMD1/PSMC3/PSMC4/PSMC5/PSMC1/PSMC2/HSP90AA1/PSMD13/PSMC6/PSMD12/PSMA2/PSMD7/PSMD11/PSMB4/PSMD14/PSMB1/CEP250/DDB1/PSMB3/ZW10/CKAP5/PPP2R1A/PSMA5/PSMA7/PSMA1/PSMA4/PSMB7/PSMD8/PSMD4/ACTR1A/PSMA6/PSMA3/DDX3X/PSMB2/CDK1/PSME3/TRIP13/PSMB6/DYNC1I2/SKP1/PRKACA/NDE1/PRMT1/DCTN2/RPS27L/TFDP1/CNOT1/PLK1
GO:0043687	post-translational protein modification	5.84123E-13	43	PSMD2/PSMD1/PSMC3/PSMC4/PSMC5/PSMC1/PSMC2/PSMD13/PSMC6/PSMD12/PSMA2/PSMD7/PSMD11/PSMB4/PSMD14/PSMB1/HSP90B1/DDB1/PSMB3/PSMA5/PSMA7/PSMA1/STT3B/PSMA4/PSMB7/PSMD8/PSMD4/PSMA6/PSMA3/RCN1/FBXW5/PSMB2/PSME3/ELOC/CKAP4/PSMB6/SKP1/APLP2/CALU/P3H1/CRTAP/CCDC8/CCN1
GO:0002758	innate immune response-activating signal transduction	6.50291E-13	39	PSMD2/OTUD4/PSMD1/PSMC3/PSMC4/PSMC5/PSMC1/PSMC2/PSMD13/PSMC6/PSMD12/NPLOC4/MAP3K7/PSMA2/PSMD7/PSMD11/PSMB4/PSMD14/PSMB1/HSP90B1/PSMB3/TAB2/TAB3/PSMA5/PSMA7/PSMA1/PSMA4/PSMB7/PSMD8/PSMD4/PSMA6/PSMA3/UFD1/PSMB2/PSME3/PSMB6/SKP1/PRKACA/CAV1

GO:0000377	RNA splicing, via transesterification reactions with bulged adenosine as nucleophile	6.69696E-13	44	PRMT5/PRPF31/WDR77/SNRNP200/HNRNPK/DDX20/CLNS1A/PRPF8/GEMIN4/SF3B1/EFTUD2/HNRNPF/HNRNPU/SF3B3/PABPC1/STRAP/HNRNPM/THRAP3/PCBP1/PCBP2/HNRNPL/AAR2/PRDX6/SMN1/SNRPD2/SF3B2/SNRPB/HNRNPA1/DHX9/SNRNP70/HNRNPA0/SNRPD3/SNRPA1/GEMIN5/PTBP1/GEMIN6/SF3B6/DHX15/HNRNPD/NSRP1/SNRPF/SNRPG/SNRPE/PABPN1
GO:0000398	mRNA splicing, via spliceosome	6.69696E-13	44	PRMT5/PRPF31/WDR77/SNRNP200/HNRNPK/DDX20/CLNS1A/PRPF8/GEMIN4/SF3B1/EFTUD2/HNRNPF/HNRNPU/SF3B3/PABPC1/STRAP/HNRNPM/THRAP3/PCBP1/PCBP2/HNRNPL/AAR2/PRDX6/SMN1/SNRPD2/SF3B2/SNRPB/HNRNPA1/DHX9/SNRNP70/HNRNPA0/SNRPD3/SNRPA1/GEMIN5/PTBP1/GEMIN6/SF3B6/DHX15/HNRNPD/NSRP1/SNRPF/SNRPG/SNRPE/PABPN1
GO:0000375	RNA splicing, via transesterification reactions	8.99005E-13	44	PRMT5/PRPF31/WDR77/SNRNP200/HNRNPK/DDX20/CLNS1A/PRPF8/GEMIN4/SF3B1/EFTUD2/HNRNPF/HNRNPU/SF3B3/PABPC1/STRAP/HNRNPM/THRAP3/PCBP1/PCBP2/HNRNPL/AAR2/PRDX6/SMN1/SNRPD2/SF3B2/SNRPB/HNRNPA1/DHX9/SNRNP70/HNRNPA0/SNRPD3/SNRPA1/GEMIN5/PTBP1/GEMIN6/SF3B6/DHX15/HNRNPD/NSRP1/SNRPF/SNRPG/SNRPE/PABPN1
GO:0045088	regulation of innate immune response	9.56499E-13	48	PRKDC/PSMD2/OTUD4/PSMD1/PSMC3/HSP90AB1/PSMC4/PSMC5/PSMC1/PSMC2/PSMD13/PSMC6/PSMD12/NPLOC4/MAP3K7/PSMA2/PSMD7/PSMD11/PSMB4/PSMD14/PSMB1/HSP90B1/PSMB3/TAB2/TAB3/PSMA5/PSMA7/PSMA1/PSMA4/NONO/PSMB7/PSMD8/PLSCR1/PSMD4/JAK1/PSMA6/PSMA3/UFD1/PSMB2/DHX9/RPS19/PSME3/MATR3/PSMB6/SKP1/PRKACA/CAV1/FAM3A
GO:1905330	regulation of morphogenesis of an epithelium	2.8998E-12	30	PSMD2/PSMD1/PSMC3/PSMC4/PSMC5/PSMC1/PSMC2/PSMD13/PSMC6/PSMD12/PSMA2/PSMD7/PSMD11/PSMB4/PSMD14/PSMB1/PSMB3/PSMA5/CLTC/PSMA7/PSMA1/PSMA4/PSMB7/PSMD8/PSMD4/PSMA6/PSMA3/PSMB2/PSME3/PSMB6
GO:0071456	cellular response to hypoxia	2.92239E-12	32	PSMD2/PSMD1/PSMC3/PSMC4/PSMC5/PSMC1/PSMC2/PSMD13/PSMC6/PSMD12/PSMA2/PSMD7/PSMD11/PSMB4/PSMD14/PSMB1/OPA1/PSMB3/PSMA5/PSMA7/PSMA1/PSMA4/PSMB7/PSMD8/PSMD4/PSMA6/PSMA3/PSMB2/PSME3/ELOC/PSMB6/STUB1
GO:0000387	spliceosomal snRNP assembly	3.31946E-12	18	PRMT5/PRPF31/WDR77/DDX20/CLNS1A/PRPF8/GEMIN4/STRAP/AAR2/SMN1/SNRPD2/SNRPB/SNRPD3/GEMIN5/GEMIN6/SNRPF/SNRPG/SNRPE
GO:0030178	negative regulation of Wnt signaling pathway	4.46651E-12	32	PSMD2/PSMD1/PSMC3/PSMC4/PSMC5/PSMC1/PSMC2/PSMD13/PSMC6/PSMD12/PSMA2/PSMD7/PSMD11/PSMB4/PSMD14/PSMB1/EMD/PSMB3/PSMA5/RACK1/PSMA7/PSMA1/PSMA4/PSMB7/PSMD8/PSMD4/PSMA6/PSMA3/PSMB2/PSME3/PSMB6/CAV1

GO:0045930	negative regulation of mitotic cell cycle	8.67581E-12	40	PRKDC/PSMD2/PSMD1/PSMC3/PSMC4/PSMC5/PSMC1/PSMC2/PSMD13/PSMC6/PSMD12/PSMA2/PSMD7/PSMD11/PSMB4/PSMD14/PSMB1/PSMB3/ZW10/PSMA5/PSMA7/PSMA1/PSMA4/PSMB7/PSMD8/PSMD4/PSMA6/PSMA3/PSMB2/CDK1/PSME3/TRIP13/PSMB6/SKP1/RPL24/PRMT1/RPS27L/TFDP1/CNOT1/PLK1
GO:0010499	proteasomal ubiquitin-independent protein catabolic process	9.4614E-12	13	PSMA2/PSMB4/PSMB1/PSMB3/PSMA5/PSMA7/PSMA1/PSMA4/PSMB7/PSMA6/PSMA3/PSMB2/PSMB6
GO:0051170	import into nucleus	1.21594E-11	28	IPO8/IPO5/KPNB1/FLNA/CSE1L/NUP188/DDX20/TRIM28/RANBP2/GEMIN4/NUP93/RANBP6/NF1/UBR5/RPL23/SMN1/SNRPD2/SNRPB/HNRNPA1/CHP1/RAN/SNRPD3/GEMIN5/TARDBP/GEMIN6/SNRPF/SNRPG/SNRPE
GO:0070482	response to oxygen levels	1.40312E-11	43	PSMD2/PSMD1/PSMC3/PSMC4/PSMC5/PSMC1/PSMC2/PSMD13/PSMC6/PSMD12/PSMA2/PSMD7/PSMD11/PSMB4/PSMD14/PSMB1/OPA1/HSP90B1/PSMB3/PSMA5/PSMA7/PSMA1/ATP6AP1/NF1/PSMA4/PSMB7/PSMD8/NAMPT/PLOD1/PSMD4/SCFD1/AIFM1/PSMA6/PSMA3/PSMB2/PSME3/ELOC/PSMB6/STUB1/ATP6V1A/TM9SF4/SLC2A1/CAV1
GO:0042180	cellular ketone metabolic process	1.45974E-11	34	PSMD2/PSMD1/PSMC3/PSMC4/PSMC5/PSMC1/PSMC2/PSMD13/PSMC6/PSMD12/ERLIN2/NMT1/PSMA2/PSMD7/PSMD11/PSMB4/PSMD14/ERLIN1/PSMB1/PSMB3/PSMA5/PSMA7/PSMA1/PSMA4/PSMB7/PSMD8/PSMD4/NDUFA9/PSMA6/PSMA3/PSMB2/PSME3/PSMB6/CAV1
GO:0030111	regulation of Wnt signaling pathway	1.93961E-11	41	VCP/PSMD2/PSMD1/PSMC3/PSMC4/PSMC5/PSMC1/PSMC2/PSMD13/PSMC6/PSMD12/PSMA2/PSMD7/PSMD11/PSMB4/PSMD14/PSMB1/SPIN1/EMD/PSMB3/PPP2R1A/PSMA5/RACK1/PSMA7/PSMA1/UBR5/PSMA4/ATP6AP2/PSMB7/PSMD8/PSMD4/PSMA6/PSMA3/DDX3X/PSMB2/PSME3/PPP1CA/PSMB6/CAV1/SMARCA4/WLS
GO:0060828	regulation of canonical Wnt signaling pathway	3.33987E-11	36	VCP/PSMD2/PSMD1/PSMC3/PSMC4/PSMC5/PSMC1/PSMC2/PSMD13/PSMC6/PSMD12/PSMA2/PSMD7/PSMD11/PSMB4/PSMD14/PSMB1/EMD/PSMB3/PSMA5/PSMA7/PSMA1/UBR5/PSMA4/PSMB7/PSMD8/PSMD4/PSMA6/PSMA3/DDX3X/PSMB2/PSME3/PPP1CA/PSMB6/CAV1/WLS
GO:0036293	response to decreased oxygen levels	3.7432E-11	41	PSMD2/PSMD1/PSMC3/PSMC4/PSMC5/PSMC1/PSMC2/PSMD13/PSMC6/PSMD12/PSMA2/PSMD7/PSMD11/PSMB4/PSMD14/PSMB1/OPA1/HSP90B1/PSMB3/PSMA5/PSMA7/PSMA1/NF1/PSMA4/PSMB7/PSMD8/NAMPT/PLOD1/PSMD4/SCFD1/AIFM1/PSMA6/PSMA3/PSMB2/PSME3/ELOC/PSMB6/STUB1/TM9SF4/SLC2A1/CAV1
GO:0036503	ERAD pathway	5.58E-11	22	VCP/HSPA5/BAG6/FAF2/PSMC6/ECPAS/NPLOC4/ERLIN2/ERLIN1/HSP90B1/UBAC2/STT3B/GET4/CCDC47/AMFR/AUP1/HM13/UFD1/STUB1/CAV1/DERL1/SEC61B
GO:0048863	stem cell differentiation	2.53264E-10	33	PSMD2/PSMD1/PSMC3/PSMC4/PSMC5/PSMC1/PSMC2/PSMD13/PSMC6/PSMD12/PSMA2/PSMD7/PSMD11/PSMB4/PSMD14/HNRNPU/PSMB1/PSMB3/PSMA5/PSMA7/PSMA1/PSMA4/PSMB7/PSMD8/PSMD4/PSMA6/PSMA3/PSMB2/PSME3/PSMB6/CFL1/CORO1C/SEMA3C

GO:0001666	response to hypoxia	3.27648E-10	39	PSMD2/PSMD1/PSMC3/PSMC4/PSMC5/PSMC1/PSMC2/PSMD13/PSMC6/PSMD12/PSMA2/PSMD7/PSMD11/PSMB4/PSMD14/PSMB1/OPA1/HSP90B1/PSMB3/PSMA5/PSMA7/PSMA1/NF1/PSMA4/PSMB7/PSMD8/PLOD1/PSMD4/SCFD1/PSMA6/PSMA3/PSMB2/PSME3/ELOC/PSMB6/STUB1/TM9SF4/SLC2A1/CAV1
GO:0010256	endomembrane system organization	5.80092E-10	43	PRMT5/ATP2A2/SPTBN1/MYH9/GOLGA2/BAG6/GCC2/TMEM43/NPLOC4/LMAN1/NUP93/EMD/ZW10/PPP2R1A/SQSTM1/AKAP8L/TMED10/GET4/PLSCR1/CCDC47/SUN2/ARL1/TOR1AIP1/SEC61A1/UBL4A/CDK1/CAMSAP3/LMAN2/TMEM33/ZMPSTE24/TMED9/CLU/RAB10/RTN3/TARDBP/STX18/TMED5/CAV1/ARHGAP21/CORO1C/RAB7A/TMED1/PLK1
GO:0071356	cellular response to tumor necrosis factor	1.67336E-09	34	PSMD2/PSMD1/PSMC3/PSMC4/PSMC5/PSMC1/PSMC2/PSMD13/PSMC6/PSMD12/PRPF8/PSMA2/PSMD7/PSMD11/PSMB4/PSMD14/PSMB1/PSMB3/PSMA5/RACK1/PSMA7/PSMA1/PSMA4/PSMB7/PSMD8/PSMD4/PSMA6/PSMA3/SMPD4/PSMB2/DHX9/PSME3/PSMB6/AKAP12
GO:0000209	protein polyubiquitination	2.06924E-09	35	HUWE1/PSMD2/PSMD1/PSMC3/PSMC4/PSMC5/PSMC1/PSMC2/PSMD13/PSMC6/PSMD12/PSMA2/PSMD7/PSMD11/PSMB4/PSMD14/PSMB1/PSMB3/PSMA5/PSMA7/PSMA1/UBR5/PSMA4/PSMB7/PSMD8/PSMD4/AMFR/PSMA6/PSMA3/FBXW5/PSMB2/PSME3/PSMB6/SKP1/STUB1
GO:0050851	antigen receptor-mediated signaling pathway	3.63148E-09	35	PSMD2/PSMD1/PSMC3/PSMC4/PSMC5/PSMC1/PSMC2/PSMD13/PSMC6/PSMD12/MAP3K7/PSMA2/PSMD7/PSMD11/PSMB4/PSMD14/STOML2/PSMB1/PSMB3/TAB2/PSMA5/PSMA7/PSMA1/PSMA4/PSMB7/PSMD8/PSMD4/PSMA6/PSMA3/PSMB2/PSME3/PSMB6/SKP1/EIF2B3/EIF2B5
GO:0060070	canonical Wnt signaling pathway	4.20633E-09	36	VCP/PSMD2/PSMD1/PSMC3/PSMC4/PSMC5/PSMC1/PSMC2/PSMD13/PSMC6/PSMD12/PSMA2/PSMD7/PSMD11/PSMB4/PSMD14/PSMB1/EMD/PSMB3/PSMA5/PSMA7/PSMA1/UBR5/PSMA4/PSMB7/PSMD8/PSMD4/PSMA6/PSMA3/DDX3X/PSMB2/PSME3/PPP1CA/PSMB6/CAV1/WLS
GO:2000027	regulation of animal organ morphogenesis	5.21407E-09	31	PSMD2/PSMD1/PSMC3/PSMC4/PSMC5/PSMC1/PSMC2/PSMD13/PSMC6/PSMD12/PSMA2/PSMD7/PSMD11/PSMB4/PSMD14/PSMB1/PSMB3/PSMA5/CLTC/PSMA7/PSMA1/PSMA4/PSMB7/PSMD8/PSMD4/PSMA6/PSMA3/PSMB2/PSME3/PSMB6/SEMA3C
GO:0030433	ubiquitin-dependent ERAD pathway	5.87243E-09	18	VCP/HSPA5/BAG6/FAF2/PSMC6/ECPAS/NPLOC4/ERLIN2/ERLIN1/HSP90B1/STT3B/CCDC47/AMFR/AUP1/STUB1/CAV1/DERL1/SEC61B
GO:0034612	response to tumor necrosis factor	1.22524E-08	34	PSMD2/PSMD1/PSMC3/PSMC4/PSMC5/PSMC1/PSMC2/PSMD13/PSMC6/PSMD12/PRPF8/PSMA2/PSMD7/PSMD11/PSMB4/PSMD14/PSMB1/PSMB3/PSMA5/RACK1/PSMA7/PSMA1/PSMA4/PSMB7/PSMD8/PSMD4/PSMA6/PSMA3/SMPD4/PSMB2/DHX9/PSME3/PSMB6/AKAP12
GO:0002429	immune response-activating cell surface receptor signaling pathway	3.09609E-08	42	PSMD2/PSMD1/PSMC3/HSP90AB1/BAG6/PSMC4/PSMC5/PSMC1/PSMC2/HSP90AA1/PSMD13/PSMC6/PSMD12/MAP3K7/PSMA2/PSMD7/PSMD11/PSMB4/PSMD14/STOML2/PSMB1/PSMB3/TAB2/TAB3/PSMA5/PSMA7/PSMA1/PSMA4/PSMB7/PSMD8/PLSCR1/PSMD4/PSMA6/PSMA3/ELMO2/PSMB2/PSME3/PSMB6/SKP1/PRKACA/EIF2B3/EIF2B5

GO:0006457	protein folding	5.48967E-08	28	VCP/HSPA5/CANX/HSP90AB1/TCP1/DNAJA2/PSMC1/DNAJC7/HSP90AA1/LMAN1/RANBP2/RAD23B/HSP90B1/CALR/DNAJB1/AMFR/CLGN/PDIA3/EMC2/SNRNP70/PRDX4/CLU/P3H1/MOGS/DERL1/CRTAP/PTGES3/EMC6
GO:0006520	cellular amino acid metabolic process	5.62355E-08	36	PSMD2/PSMD1/PSMC3/PSMC4/PSMC5/PSMC1/PSMC2/PSMD13/PSMC6/PSMD12/PHGDH/PSMA2/PSMD7/PSMD11/PSMB4/PSMD14/PSMB1/PSMB3/PSMA5/PSMA7/PSMA1/PSMA4/PSMB7/PSMD8/PLOD1/PSMD4/PSMA6/PSMA3/PSMB2/PSME3/AIMP1/PSMB6/GART/PRDX4/SLC1A3/CRTAP
GO:0042255	ribosome assembly	2.65631E-07	15	RPSA/RPL3/RPL6/RPS15/DDX3X/RPL5/RPL10/RPS5/RPS19/RPL12/RPL24/RPL11/RPS28/RPS27L/RPL23A
GO:0034504	protein localization to nucleus	3.40519E-07	29	IPO8/XPO1/IPO5/KPNB1/FLNA/TCP1/CSE1L/NUP188/TRIM28/RANBP2/HNRNPU/NUP93/RANBP6/NF1/UBR5/CALR/RPL23/RANGAP1/SUN2/TOR1AIP1/CDK1/OSBPL8/CHP1/SKP1/RAN/RPL11/TARDBP/TXN/PLK1
GO:0017038	protein import	4.61302E-07	25	IPO8/IPO5/KPNB1/FLNA/CSE1L/NUP188/HSP90AA1/TRIM28/RANBP2/NUP93/TOMM40/RANBP6/NF1/UBR5/RPL23/TIMM50/TIMM23B/SAMM50/CHP1/RAN/AGK/CLU/TARDBP/NDUFA13/PEX3
GO:0032527	protein exit from endoplasmic reticulum	4.77639E-07	13	VCP/GCC2/FAF2/NPLOC4/HSP90B1/UBAC2/AUP1/SAR1A/HM13/UFD1/TM9SF4/DERL1/SEC61B
GO:0042176	regulation of protein catabolic process	7.18869E-07	35	VCP/XPO1/PSMD2/FLNA/PSMD1/HSP90AB1/BAG6/PSMC4/PSMC1/PSMC2/HSP90AA1/PSMD14/RAD23B/CYP51A1/DDB1/SF3B3/UBAC2/RACK1/RPL23/RPL5/LPCAT1/PSME3/SEC22B/SNRNP70/PRKACA/CLU/OGT/RPL11/STUB1/NDUFA13/CAV1/RHBDD3/TMTC3/RAB7A/PLK1
GO:0048193	Golgi vesicle transport	1.12979E-06	34	VCP/SPTBN1/GOLGA2/GCC2/LMAN1/ZW10/RACK1/PPP6R1/TMED10/VPS13A/COPA/SCAMP3/SCFD1/ACTR1A/GOLGA3/ARL1/SAR1A/PPP6C/LMAN2/COPG1/SEC22B/DYNC1I2/ARCN1/ARF4/TMED9/CNIH4/RAB10/STX18/TMED5/EXOC4/KIF5A/DCTN2/CAPZA1/TMED1
GO:0042254	ribosome biogenesis	1.52853E-06	30	XPO1/HEATR1/MYBBP1A/GEMIN4/RPSA/RPL3/RPL6/RPS8/RPS15/DDX3X/RPL5/DDX21/RPL10/RPS5/BMS1/RPS19/RPL27/RAN/RPL12/RPL14/FBL/RPL24/RPL11/RPS28/RPS27L/RPL10A/NOM1/RPS9/RPL23A/RPL35
GO:0006888	ER to Golgi vesicle-mediated transport	1.79805E-06	25	VCP/SPTBN1/GOLGA2/LMAN1/ZW10/PPP6R1/TMED10/COPA/SCFD1/ACTR1A/SAR1A/PPP6C/LMAN2/COPG1/SEC22B/DYNC1I2/ARCN1/ARF4/TMED9/CNIH4/STX18/TMED5/DCTN2/CAPZA1/TMED1
GO:0044766	multi-organism transport	2.47147E-06	15	XPO1/KPNB1/TCP1/NUP188/NUP160/RANBP2/NUP93/NUP210/RAN/NUP37/CAV1/DYNLT1/RAE1/RAB7A/THOC3
GO:1902579	multi-organism localization	2.47147E-06	15	XPO1/KPNB1/TCP1/NUP188/NUP160/RANBP2/NUP93/NUP210/RAN/NUP37/CAV1/DYNLT1/RAE1/RAB7A/THOC3
GO:0019058	viral life cycle	4.01884E-06	31	VCP/XPO1/KPNB1/HSP90AB1/NUP188/NUP160/TRIM28/RANBP2/STOM/NUP93/HACD3/RPSA/TFRC/DDB1/PABPC1/SLC1A5/PLSCR1/PCBP1/PCBP2/NUP210/ITGB1/PI4KA/ZC3HAV1/DDX3X/CDK1/RAN/NUP37/CAV1/DYNLT1/RAE1/RAB7A
GO:0030970	retrograde protein transport, ER to cytosol	7.51847E-06	10	VCP/FAF2/NPLOC4/HSP90B1/UBAC2/AUP1/HM13/UFD1/DERL1/SEC61B

GO:1903513	endoplasmic reticulum to cytosol transport	7.51847E-06	10	VCP/FAF2/NPLOC4/HSP90B1/UBAC2/AUP1/HM13/UFD1/DERL1/SEC61B
GO:0051168	nuclear export	8.03813E-06	23	XPO1/XPOT/CSE1L/NUP188/NUP160/RANBP2/NUP93/EMD/AKAP8L/CALR/RANGAP1/RPS15/NUP210/HNRNPA1/DHX9/CHP1/RAN/PRKACA/NUP37/RAE1/THOC3/TXN/PABPN1
GO:0001732	formation of cytoplasmic translation initiation complex	9.20188E-06	8	EIF3B/EIF3A/EIF3I/EIF3M/EIF3F/EIF3H/EIF3G/EIF5
GO:0043312	neutrophil degranulation	1.1076E-05	38	HUWE1/VCP/PSMD2/KPNB1/PSMD1/PSMC3/HSP90AB1/PSMC2/HSP90AA1/DDOST/FAF2/PSMD13/PSMD12/STOM/EEF2/PSMA2/PSMD7/PSMD11/PSMD14/UBR4/ATAD3B/PSMB1/PSMA5/DSP/ATP6AP2/PSMB7/PGRMC1/PRDX6/DDX3X/IGF2R/LPCAT1/IMPDH2/CKAP4/PRDX4/RAB10/DYNLT1/RAB7A/MANBA
GO:0002283	neutrophil activation involved in immune response	1.31228E-05	38	HUWE1/VCP/PSMD2/KPNB1/PSMD1/PSMC3/HSP90AB1/PSMC2/HSP90AA1/DDOST/FAF2/PSMD13/PSMD12/STOM/EEF2/PSMA2/PSMD7/PSMD11/PSMD14/UBR4/ATAD3B/PSMB1/PSMA5/DSP/ATP6AP2/PSMB7/PGRMC1/PRDX6/DDX3X/IGF2R/LPCAT1/IMPDH2/CKAP4/PRDX4/RAB10/DYNLT1/RAB7A/MANBA
GO:0042119	neutrophil activation	2.28526E-05	38	HUWE1/VCP/PSMD2/KPNB1/PSMD1/PSMC3/HSP90AB1/PSMC2/HSP90AA1/DDOST/FAF2/PSMD13/PSMD12/STOM/EEF2/PSMA2/PSMD7/PSMD11/PSMD14/UBR4/ATAD3B/PSMB1/PSMA5/DSP/ATP6AP2/PSMB7/PGRMC1/PRDX6/DDX3X/IGF2R/LPCAT1/IMPDH2/CKAP4/PRDX4/RAB10/DYNLT1/RAB7A/MANBA
GO:0002446	neutrophil mediated immunity	2.41278E-05	38	HUWE1/VCP/PSMD2/KPNB1/PSMD1/PSMC3/HSP90AB1/PSMC2/HSP90AA1/DDOST/FAF2/PSMD13/PSMD12/STOM/EEF2/PSMA2/PSMD7/PSMD11/PSMD14/UBR4/ATAD3B/PSMB1/PSMA5/DSP/ATP6AP2/PSMB7/PGRMC1/PRDX6/DDX3X/IGF2R/LPCAT1/IMPDH2/CKAP4/PRDX4/RAB10/DYNLT1/RAB7A/MANBA
GO:0009408	response to heat	3.58402E-05	21	VCP/HSPA5/ATP2A2/HSP90AB1/DNAJA2/DNAJC7/NUP188/HSP90AA1/NUP160/RANBP2/NUP93/NF1/DNAJB1/NUP210/PRKACA/NUP37/STUB1/EIF2B3/RAE1/EIF2B5/PTGES3
GO:0002183	cytoplasmic translational initiation	4.35612E-05	10	EIF4B/EIF3B/EIF3A/EIF3I/EIF3M/EIF3F/EIF2S3/EIF3H/EIF3G/EIF5
GO:0006611	protein export from nucleus	4.84856E-05	21	XPO1/XPOT/CSE1L/NUP188/NUP160/RANBP2/NUP93/EMD/AKAP8L/CALR/RANGAP1/RPS15/NUP210/CHP1/RAN/PRKACA/NUP37/RAE1/THOC3/TXN/PABPN1
GO:0034605	cellular response to heat	9.03757E-05	18	VCP/HSPA5/ATP2A2/HSP90AB1/DNAJC7/NUP188/HSP90AA1/NUP160/RANBP2/NUP93/NF1/DNAJB1/NUP210/PRKACA/NUP37/STUB1/RAE1/PTGES3
GO:0019081	viral translation	9.5669E-05	7	EIF3B/EIF3A/PCBP2/EIF3F/DHX9/PTBP1/EIF3G
GO:0033157	regulation of intracellular protein transport	0.000137032	23	HUWE1/XPO1/IPO5/FLNA/GCC2/LMAN1/TRIM28/STOM/EMD/UBAC2/AKAP8L/NF1/UBR5/HAX1/RANGAP1/SAR1A/CHP1/RAN/MFF/PRKACA/TARDBP/TM9SF4/TXN

GO:0046794	transport of virus	0.000175337	12	XPO1/KPNB1/NUP188/NUP160/RANBP2/NUP93/NUP210/RAN/NUP37/CAV1/DYNLT1/RAE1
GO:1903706	regulation of hemopoiesis	0.000219514	35	PSMD2/PSMD1/PSMC3/PSMC4/PSMC5/PSMC1/PSMC2/PSMD13/PSMC6/PSMD12/TNRC6B/PSMA2/PSMD7/PSMD11/PSMB4/PSMD14/PSMB1/CLPTM1/PSMB3/PSMA5/PSMA7/PSMA1/ATP6AP1/NF1/PSMA4/HAX1/PSMB7/PSMD8/PSMD4/PSMA6/PSMA3/PSMB2/PSME3/PSMB6/PRMT1
GO:0034975	protein folding in endoplasmic reticulum	0.000304111	7	HSPA5/CANX/HSP90B1/CALR/PDIA3/EMC2/EMC6
GO:0035966	response to topologically incorrect protein	0.000310601	21	VCP/HSPA5/CANX/HSP90AB1/BAG6/HSP90AA1/FAF2/HSP90B1/RACK1/STT3B/CALR/DNAJB1/AMFR/CLGN/UFD1/SRPRB/TMEM33/SSR1/CLU/STUB1/DERL1
GO:0006998	nuclear envelope organization	0.000345663	11	TMEM43/NUP93/EMD/PPP2R1A/AKAP8L/SUN2/TOR1AIP1/CDK1/ZMPSTE24/TARDBP/PLK1
GO:0046822	regulation of nucleocytoplasmic transport	0.000378051	15	XPO1/IPO5/FLNA/TRIM28/EMD/AKAP8L/NF1/UBR5/RANGAP1/DHX9/CHP1/RAN/PRKACA/TARDBP/TXN
GO:0009152	purine ribonucleotide biosynthetic process	0.000505927	25	VCP/ATP5F1A/FASN/NUP188/NUP160/RANBP2/STOML2/NUP93/PFKFB3/SLC25A1/PDHB/HSD17B12/NUP210/IMPDH2/PPT1/NUP37/GART/OGT/CYC1/SCD/DLAT/ATP5F1D/PDHA1/ATP5PD/RAE1
GO:0007007	inner mitochondrial membrane organization	0.000646906	10	IMMT/ATP5F1A/OPA1/DNAJC11/CHCHD3/SAMM50/AGK/ATP5F1D/NDUFA13/ATP5PD
GO:0006409	tRNA export from nucleus	0.000651581	9	XPOT/NUP188/NUP160/RANBP2/NUP93/NUP210/RAN/NUP37/RAE1
GO:0071431	tRNA-containing ribonucleoprotein complex export from nucleus	0.000651581	9	XPOT/NUP188/NUP160/RANBP2/NUP93/NUP210/RAN/NUP37/RAE1
GO:0007029	endoplasmic reticulum organization	0.000934794	11	BAG6/LMAN1/GET4/CCDC47/SEC61A1/UBL4A/LMAN2/TMEM33/RAB10/RTN3/STX18
GO:0075733	intracellular transport of virus	0.000934794	11	XPO1/KPNB1/NUP188/NUP160/RANBP2/NUP93/NUP210/RAN/NUP37/DYNLT1/RAE1
GO:1903050	regulation of proteolysis involved in cellular protein catabolic process	0.001066664	21	VCP/HSP90AB1/BAG6/PSMC4/PSMC1/PSMC2/PSMD14/RAD23B/UBAC2/RACK1/RPL23/RPL5/PSME3/PRKACA/CLU/OGT/RPL11/STUB1/CAV1/TMTC3/PLK1
GO:0022406	membrane docking	0.001067298	19	HSP90AA1/CEP250/CKAP5/PPP2R1A/SCFD1/ACTR1A/CDK1/CEP83/CHP1/DYNC1I2/ESYT2/PRKACA/NDE1/IQCB1/RAB10/EXOC4/DCTN2/RAB7A/PLK1
GO:0051031	tRNA transport	0.001113525	9	XPOT/NUP188/NUP160/RANBP2/NUP93/NUP210/RAN/NUP37/RAE1
GO:0009260	ribonucleotide biosynthetic process	0.001197859	25	VCP/ATP5F1A/FASN/NUP188/NUP160/RANBP2/STOML2/NUP93/PFKFB3/SLC25A1/PDHB/HSD17B12/NUP210/IMPDH2/PPT1/NUP37/GART/OGT/CYC1/SCD/DLAT/ATP5F1D/PDHA1/ATP5PD/RAE1
GO:0009205	purine ribonucleoside triphosphate metabolic process	0.001272874	27	VCP/ATP5F1A/NUP188/NUP160/RANBP2/STOML2/NUP93/PFKFB3/OPA1/NDUFA9/NUP210/NDUFS2/CDK1/IMPDH2/RAN/NUP37/NDUFS3/OGT/CYC1/SDHA/ATP6V1A/ATP5F1D/NDUFA13/NDUFB10/ATP5PD/RAE1/NDUFA8

GO:0061136	regulation of proteasomal protein catabolic process	0.001641862	19	VCP/HSP90AB1/BAG6/PSMC4/PSMC1/PSMC2/PSMD14/RAD23B/UBAC2/RACK1/PSME3/PRKACA/CLU/OGT/RPL11/STUB1/CAV1/TMTC3/PLK1
GO:0009167	purine ribonucleoside monophosphate metabolic process	0.001705084	27	VCP/ATP5F1A/NUP188/NUP160/RANBP2/STOML2/NUP93/PFKFB3/NDUFA9/NUP210/NDUFS2/CDK1/IMPDH2/NUP37/GART/NDUFS3/OGT/CYC1/NT5C2/SDHA/ATP6V1A/ATP5F1D/NDUFA13/NDUFB10/ATP5PD/RAE1/NDUFA8
GO:0009126	purine nucleoside monophosphate metabolic process	0.001806065	27	VCP/ATP5F1A/NUP188/NUP160/RANBP2/STOML2/NUP93/PFKFB3/NDUFA9/NUP210/NDUFS2/CDK1/IMPDH2/NUP37/GART/NDUFS3/OGT/CYC1/NT5C2/SDHA/ATP6V1A/ATP5F1D/NDUFA13/NDUFB10/ATP5PD/RAE1/NDUFA8
GO:0009199	ribonucleoside triphosphate metabolic process	0.001806065	27	VCP/ATP5F1A/NUP188/NUP160/RANBP2/STOML2/NUP93/PFKFB3/OPA1/NDUFA9/NUP210/NDUFS2/CDK1/IMPDH2/RAN/NUP37/NDUFS3/OGT/CYC1/SDHA/ATP6V1A/ATP5F1D/NDUFA13/NDUFB10/ATP5PD/RAE1/NDUFA8
GO:0097064	ncRNA export from nucleus	0.001835331	9	XPOT/NUP188/NUP160/RANBP2/NUP93/NUP210/RAN/NUP37/RAE1
GO:0006164	purine nucleotide biosynthetic process	0.00186174	25	VCP/ATP5F1A/FASN/NUP188/NUP160/RANBP2/STOML2/NUP93/PFKFB3/SLC25A1/PDHB/HSD17B12/NUP210/IMPDH2/PPT1/NUP37/GART/OGT/CYC1/SCD/DLAT/ATP5F1D/PDHA1/ATP5PD/RAE1
GO:0046390	ribose phosphate biosynthetic process	0.00186174	25	VCP/ATP5F1A/FASN/NUP188/NUP160/RANBP2/STOML2/NUP93/PFKFB3/SLC25A1/PDHB/HSD17B12/NUP210/IMPDH2/PPT1/NUP37/GART/OGT/CYC1/SCD/DLAT/ATP5F1D/PDHA1/ATP5PD/RAE1
GO:0009144	purine nucleoside triphosphate metabolic process	0.001910411	27	VCP/ATP5F1A/NUP188/NUP160/RANBP2/STOML2/NUP93/PFKFB3/OPA1/NDUFA9/NUP210/NDUFS2/CDK1/IMPDH2/RAN/NUP37/NDUFS3/OGT/CYC1/SDHA/ATP6V1A/ATP5F1D/NDUFA13/NDUFB10/ATP5PD/RAE1/NDUFA8
GO:0006405	RNA export from nucleus	0.002243885	16	XPO1/XPOT/NUP188/NUP160/RANBP2/NUP93/AKAP8L/RPS15/NUP210/HNRNPA1/DHX9/RAN/NUP37/RAE1/THOC3/PABPN1
GO:0006695	cholesterol biosynthetic process	0.00226254	12	KPNB1/FASN/ERLIN2/ERLIN1/CYP51A1/LBR/DHCR7/FDFT1/RAN/DHCR24/SCD/SQLE
GO:1903829	positive regulation of cellular protein localization	0.00227402	26	HUWE1/IPO5/SPTBN1/FLNA/HSP90AB1/TCP1/NMT1/TRIM28/STOM/CEP250/EMD/SQSTM1/RACK1/UBR5/SAR1A/ITGB1/CDK1/CHP1/RAN/MFF/PRKACA/GNL3/TARDBP/TM9SF4/TFDP1/PLK1
GO:0045540	regulation of cholesterol biosynthetic process	0.002307338	10	KPNB1/FASN/ERLIN2/ERLIN1/CYP51A1/DHCR7/FDFT1/RAN/SCD/SQLE
GO:0106118	regulation of sterol biosynthetic process	0.002307338	10	KPNB1/FASN/ERLIN2/ERLIN1/CYP51A1/DHCR7/FDFT1/RAN/SCD/SQLE
GO:0031647	regulation of protein stability	0.002440396	24	PRKDC/FLNA/HSP90AB1/TCP1/BAG6/HSP90AA1/TELO2/NCLN/USP9X/GET4/CALR/RPL23/ATP1B3/RPL5/CHP1/CLU/P3H1/RPL11/TARDBP/STUB1/DERL1/CRTAP/PTGES3/PLK1
GO:1902653	secondary alcohol biosynthetic process	0.002619093	12	KPNB1/FASN/ERLIN2/ERLIN1/CYP51A1/LBR/DHCR7/FDFT1/RAN/DHCR24/SCD/SQLE
GO:1903362	regulation of cellular protein catabolic process	0.002947479	22	VCP/HSP90AB1/BAG6/PSMC4/PSMC1/PSMC2/HSP90AA1/PSMD14/RAD23B/UBAC2/RACK1/RPL23/RPL5/PSME3/PRKACA/CLU/OGT/RPL11/STUB1/CAV1/TMTC3/PLK1
GO:0000027	ribosomal large subunit assembly	0.002975837	8	RPL3/RPL6/RPL5/RPL10/RPL12/RPL24/RPL11/RPL23A

GO:0009895	negative regulation of catabolic process	0.003023147	25	FLNA/GOLGA2/HSP90AB1/BAG6/LRPPRC/HNRNPU/CYP51A1/SF3B3/PABPC1/UBAC2/TAB2/TAB3/RPL23/NAMPT/THRAP3/SCFD1/RPL5/DHX9/SEC22B/SNRNP70/HNRNPA0/OGT/RPL11/TARDBP/HNRNPD
GO:0006403	RNA localization	0.003495842	21	XPO1/XPOT/TCP1/NUP188/NUP160/RANBP2/LRPPRC/HNRNPU/NUP93/CKAP5/AKAP8L/RPS15/NUP210/HNRNPA1/DHX9/RAN/NUP37/FBL/RAE1/THOC3/PABPN1
GO:0050657	nucleic acid transport	0.00369831	19	XPO1/XPOT/NUP188/NUP160/RANBP2/LRPPRC/HNRNPU/NUP93/CKAP5/AKAP8L/RPS15/NUP210/HNRNPA1/DHX9/RAN/NUP37/RAE1/THOC3/PABPN1
GO:0050658	RNA transport	0.00369831	19	XPO1/XPOT/NUP188/NUP160/RANBP2/LRPPRC/HNRNPU/NUP93/CKAP5/AKAP8L/RPS15/NUP210/HNRNPA1/DHX9/RAN/NUP37/RAE1/THOC3/PABPN1
GO:0009161	ribonucleoside monophosphate metabolic process	0.003725158	27	VCP/ATP5F1A/NUP188/NUP160/RANBP2/STOML2/NUP93/PFKFB3/NDUFA9/NUP210/NDUFS2/CDK1/IMPDH2/NUP37/GART/NDUFS3/OGT/CYC1/NT5C2/SDHA/ATP6V1A/ATP5F1D/NDUFA13/NDUFB10/ATP5PD/RAE1/NDUFA8
GO:1903320	regulation of protein modification by small protein conjugation or removal	0.003747829	21	HUWE1/VCP/HSPA5/OTUD4/FANCI/GOLGA2/HSP90AB1/HSP90AA1/IVNS1ABP/UBR5/RPL23/RPL5/CHP1/OGT/GNL3/RPL11/STUB1/CAV1/DERL1/NDFIP1/PLK1
GO:1900034	regulation of cellular response to heat	0.004011704	12	HSP90AB1/DNAJC7/NUP188/HSP90AA1/NUP160/RANBP2/NUP93/DNAJB1/NUP210/NUP37/RAE1/PTGES3
GO:0072522	purine-containing compound biosynthetic process	0.00405553	25	VCP/ATP5F1A/FASN/NUP188/NUP160/RANBP2/STOML2/NUP93/PFKFB3/SLC25A1/PDHB/HSD17B12/NUP210/IMPDH2/PPT1/NUP37/GART/OGT/CYC1/SCD/DLAT/ATP5F1D/PDHA1/ATP5PD/RAE1
GO:0006986	response to unfolded protein	0.004073271	18	VCP/HSPA5/CANX/HSP90AB1/HSP90AA1/FAF2/HSP90B1/RACK1/STT3B/CALR/DNAJB1/AMFR/CLGN/SRPRB/TMEM33/SSR1/STUB1/DERL1
GO:0007030	Golgi organization	0.004430565	16	PRMT5/GOLGA2/GCC2/NPLOC4/LMAN1/ZW10/TMED10/ARL1/CDK1/CAMSAP3/LMAN2/TMED9/STX18/TMED5/ARHGAP21/TMED1
GO:0051236	establishment of RNA localization	0.004662704	19	XPO1/XPOT/NUP188/NUP160/RANBP2/LRPPRC/HNRNPU/NUP93/CKAP5/AKAP8L/RPS15/NUP210/HNRNPA1/DHX9/RAN/NUP37/RAE1/THOC3/PABPN1
GO:0050821	protein stabilization	0.004799309	18	FLNA/HSP90AB1/TCP1/BAG6/HSP90AA1/TELO2/NCLN/USP9X/CALR/RPL23/ATP1B3/RPL5/CHP1/CLU/P3H1/RPL11/CRTAP/PTGES3
GO:0006606	protein import into nucleus	0.004864381	16	IPO8/IPO5/KPNB1/FLNA/CSE1L/NUP188/TRIM28/RANBP2/NUP93/RANBP6/NF1/UBR5/RPL23/CHP1/RAN/TARDBP
GO:0097711	ciliary basal body-plasma membrane docking	0.00489465	13	HSP90AA1/CEP250/CKAP5/PPP2R1A/ACTR1A/CDK1/CEP83/DYNC1I2/PRKACA/NDE1/IQCB1/DCTN2/PLK1
GO:0016126	sterol biosynthetic process	0.005267919	12	KPNB1/FASN/ERLIN2/ERLIN1/CYP51A1/LBR/DHCR7/FDFT1/RAN/DHCR24/SCD/SQLE

GO:0006839	mitochondrial transport	0.005349274	22	HUWE1/ATP5F1A/HSP90AA1/LMAN1/NMT1/STOML2/OPA1/TOMM40/HAX1/TIMM50/SLC25A1/MTX2/TIMM23B/SAMM50/AGK/MFF/CYC1/TFDP1/ATP5F1D/NDUFA13/ATP5PD/DYNLT1
GO:0009141	nucleoside triphosphate metabolic process	0.00570192	27	VCP/ATP5F1A/NUP188/NUP160/RANBP2/STOML2/NUP93/PFKFB3/OPA1/NDUFA9/NUP210/NDUFS2/CDK1/IMPDH2/RAN/NUP37/NDUFS3/OGT/CYC1/SDHA/ATP6V1A/ATP5F1D/NDUFA13/NDUFB10/ATP5PD/RAE1/NDUFA8
GO:0071166	ribonucleoprotein complex localization	0.00576139	15	XPO1/XPOT/PRPF31/NUP188/NUP160/RANBP2/NUP93/AKAP8L/RPS15/NUP210/RAN/NUP37/RAE1/THOC3/PABPN1
GO:1902850	microtubule cytoskeleton organization involved in mitosis	0.00773463	15	VCP/KPNB1/FLNA/GOLGA2/HNRNPU/ZW10/CLTC/SUN2/ACTR1A/RAN/NDE1/DCTN2/DYNLT1/RAE1/PLK1
GO:0009266	response to temperature stimulus	0.008443964	21	VCP/HSPA5/ATP2A2/HSP90AB1/DNAJA2/DNAJC7/NUP188/HSP90AA1/NUP160/RANBP2/NUP93/NF1/DNAJB1/NUP210/PRKACA/NUP37/STUB1/EIF2B3/RAE1/EIF2B5/PTGES3
GO:0046034	ATP metabolic process	0.008591813	24	VCP/ATP5F1A/NUP188/NUP160/RANBP2/STOML2/NUP93/PFKFB3/NDUFA9/NUP210/NDUFS2/CDK1/NUP37/NDUFS3/OGT/CYC1/SDHA/ATP6V1A/ATP5F1D/NDUFA13/NDUFB10/ATP5PD/RAE1/NDUFA8
GO:0031330	negative regulation of cellular catabolic process	0.008885257	22	GOLGA2/HSP90AB1/BAG6/LRPPRC/HNRNPU/PABPC1/UBAC2/TAB2/TAB3/RPL23/NAMPT/THRAP3/SCFD1/RPL5/DHX9/SEC22B/SNRNP70/HNRNPA0/OGT/RPL11/TARDBP/HNRNPD
GO:0042273	ribosomal large subunit biogenesis	0.009153494	11	RPL3/RPL6/RPL5/RPL10/RPL12/RPL14/RPL24/RPL11/RPL10A/RPL23A/RPL35
GO:0006890	retrograde vesicle-mediated transport, Golgi to ER	0.010071191	12	ZW10/TMED10/COPA/SCFD1/LMAN2/COPG1/SEC22B/ARCN1/ARF4/TMED9/STX18/KIF5A
GO:0140056	organelle localization by membrane tethering	0.010084308	17	HSP90AA1/CEP250/CKAP5/PPP2R1A/SCFD1/ACTR1A/CDK1/CEP83/DYNC1I2/ESYT2/PRKACA/NDE1/IQCB1/RAB10/EXOC4/DCTN2/PLK1
GO:0006984	ER-nucleus signaling pathway	0.010156886	9	HSPA5/ATP2A2/ERLIN2/ERLIN1/HSP90B1/CALR/CCDC47/TMEM33/TMCO1
GO:0007006	mitochondrial membrane organization	0.010272485	15	IMMT/ATP5F1A/HSP90AA1/NMT1/OPA1/TIMM50/DNAJC11/CHCHD3/SAMM50/AGK/TFDP1/ATP5F1D/NDUFA13/ATP5PD/DYNLT1
GO:0075522	IRES-dependent viral translational initiation	0.010480273	5	EIF3B/EIF3A/PCBP2/EIF3F/PTBP1
GO:0007051	spindle organization	0.010921976	17	VCP/KPNB1/FLNA/MYH9/GOLGA2/HNRNPU/TUBGCP4/TUBGCP3/CKAP5/PPP2R1A/CLTC/SUN2/RAN/TUBGCP2/DCTN2/RAE1/PLK1
GO:0009123	nucleoside monophosphate metabolic process	0.011071488	27	VCP/ATP5F1A/NUP188/NUP160/RANBP2/STOML2/NUP93/PFKFB3/NDUFA9/NUP210/NDUFS2/CDK1/IMPDH2/NUP37/GART/NDUFS3/OGT/CYC1/NT5C2/SDHA/ATP6V1A/ATP5F1D/NDUFA13/NDUFB10/ATP5PD/RAE1/NDUFA8
GO:0034250	positive regulation of cellular amide metabolic process	0.011863238	16	EEF2/PABPC1/RACK1/RPS4X/DDX3X/RPL5/DHX9/EIF2S3/CLU/RPS27L/VIM/HNRNPD/RPS9/EIF2B5/CDC123/CCN1

GO:0045899	positive regulation of RNA polymerase II transcriptional preinitiation complex assembly	0.018771844	5	PSMC3/PSMC4/PSMC5/PSMC2/PSMC6
GO:0090181	regulation of cholesterol metabolic process	0.020838646	10	KPNB1/FASN/ERLIN2/ERLIN1/CYP51A1/DHCR7/FDFT1/RAN/SCD/SQLE
GO:0034248	regulation of cellular amide metabolic process	0.022452866	31	GCN1/EIF4B/EIF3B/TNRC6B/EEF2/LRPPRC/LTN1/PABPC1/RACK1/TMED10/RPS4X/CALR/DDX3X/RPL5/RPL10/DHX9/EIF2S3/PPP1CA/EIF3H/SNRNP70/GEMIN5/CLU/RPS27L/VIM/CNOT1/HNRNPD/RPS9/EIF5/EIF2B5/CDC123/CCN1
GO:0000028	ribosomal small subunit assembly	0.024676547	6	RPSA/RPS15/RPS5/RPS19/RPS28/RPS27L
GO:0009108	coenzyme biosynthetic process	0.02580087	21	FASN/NUP188/NUP160/RANBP2/NUP93/PFKFB3/NAMPT/SLC25A1/PDHB/HSD17B12/NDUFA9/NUP210/PPT1/MAT2A/NUP37/GART/OGT/SCD/DLAT/PDHA1/RAE1
GO:0071426	ribonucleoprotein complex export from nucleus	0.02617977	14	XPO1/XPOT/NUP188/NUP160/RANBP2/NUP93/AKAP8L/RPS15/NUP210/RAN/NUP37/RAE1/THOC3/PABPN1
GO:0009127	purine nucleoside monophosphate biosynthetic process	0.029490097	17	VCP/ATP5F1A/NUP188/NUP160/RANBP2/STOML2/NUP93/PFKFB3/NUP210/IMPDH2/NUP37/GART/OGT/CYC1/ATP5F1D/ATP5PD/RAE1
GO:0009168	purine ribonucleoside monophosphate biosynthetic process	0.029490097	17	VCP/ATP5F1A/NUP188/NUP160/RANBP2/STOML2/NUP93/PFKFB3/NUP210/IMPDH2/NUP37/GART/OGT/CYC1/ATP5F1D/ATP5PD/RAE1
GO:0031396	regulation of protein ubiquitination	0.032952967	18	HUWE1/HSPA5/FANCI/GOLGA2/HSP90AB1/HSP90AA1/IVNS1ABP/UBR5/RPL23/RPL5/CHP1/OGT/RPL11/STUB1/CAV1/DERL1/NDFIP1/PLK1
GO:0032386	regulation of intracellular transport	0.035722399	28	HUWE1/XPO1/IPO5/ATP2A2/FLNA/MAP1B/GCC2/LMAN1/TRIM28/STOM/EMD/UBAC2/AKAP8L/NF1/UBR5/HAX1/RANGAP1/SCFD1/SAR1A/DHX9/CHP1/RAN/MFF/PRKACA/TARDBP/STX18/TM9SF4/TXN
GO:0042274	ribosomal small subunit biogenesis	0.036672899	10	HEATR1/RPSA/RPS8/RPS15/RPS5/BMS1/RPS19/RPS28/RPS27L/NOM1
GO:0043900	regulation of multi-organism process	0.045215419	27	PSMC3/NPLOC4/TRIM28/STOM/HACD3/DDB1/PABPC1/PPP2R1A/PPM1B/CALR/PLSCR1/PCBP2/ZC3HAV1/EXOC7/DDX3X/IGF2R/UFD1/DHX9/ZMPSTE24/PRDX4/CFL1/TARDBP/CAV1/SMARCA4/DYNLT1/FAM3A/RAB7A
GO:0009206	purine ribonucleoside triphosphate biosynthetic process	0.049355226	16	VCP/ATP5F1A/NUP188/NUP160/RANBP2/STOML2/NUP93/PFKFB3/NUP210/IMPDH2/NUP37/OGT/CYC1/ATP5F1D/ATP5PD/RAE1
GO:0043248	proteasome assembly	0.049846018	5	PSMD13/ECPAS/PSMD11/PSMD4/ADRM1
